# The brain acid‐soluble protein 1 (BASP1) interferes with the oncogenic capacity of MYC and its binding to calmodulin

**DOI:** 10.1002/1878-0261.12636

**Published:** 2020-01-30

**Authors:** Markus Hartl, Kane Puglisi, Andrea Nist, Philipp Raffeiner, Klaus Bister

**Affiliations:** ^1^ Institute of Biochemistry and Center for Molecular Biosciences (CMBI) University of Innsbruck Austria; ^2^ Present address: Genomics Core Facility Philipps University of Marburg Germany; ^3^ Present address: Department of Molecular Medicine Scripps Research La Jolla CA USA

**Keywords:** calcium signaling, cancer, protein stability, transcription factor, tumor suppressor

## Abstract

The MYC protein is a transcription factor with oncogenic potential controlling fundamental cellular processes such as cell proliferation, metabolism, differentiation, and apoptosis. The *MYC* gene is a major cancer driver, and elevated MYC protein levels are a hallmark of most human cancers. We have previously shown that the brain acid‐soluble protein 1 gene (*BASP1*) is specifically downregulated by the v‐*myc* oncogene and that ectopic *BASP1* expression inhibits v‐*myc*‐induced cell transformation. The 11‐amino acid effector domain of the BASP1 protein interacts with the calcium sensor calmodulin (CaM) and is mainly responsible for this inhibitory function. We also reported recently that CaM interacts with all MYC variant proteins and that ectopic CaM increases the transactivation and transformation potential of the v‐Myc protein. Here, we show that the presence of excess BASP1 or of a synthetic BASP1 effector domain peptide leads to displacement of v‐Myc from CaM. The protein stability of v‐Myc is decreased in cells co‐expressing v‐Myc and BASP1, which may account for the inhibition of v‐Myc. Furthermore, suppression of v‐Myc‐triggered transcriptional activation and cell transformation is compensated by ectopic CaM, suggesting that BASP1‐mediated withdrawal of CaM from v‐Myc is a crucial event in the inhibition. In view of the tumor‐suppressive role of BASP1 which was recently also reported for human cancer, small compounds or peptides based on the BASP1 effector domain could be used in drug development strategies aimed at tumors with high MYC expression.

## Graphical abstract

The MYC oncoprotein is a major cancer driver. We present evidence for a functional connection between MYC, the MYC target BASP1, and calmodulin (CaM). We propose that BASP1 competes with MYC for Ca^2+^‐dependent CaM binding leading to MYC destabilization as molecular mechanism of BASP1‐induced MYC inhibition. Enhanced MYC degradation leads to less nuclear entry, thereby preventing deregulation of transcriptional targets.

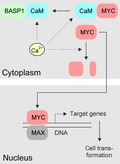

**Abbreviations**
AML: acute myeloid leukemia
AR: androgen receptor
ASV17: avian sarcoma virus 17
BASP1: brain acid‐soluble protein 1
CALM: calmodulin (gene)
CaM: calmodulin (protein)
CaM‐ag.: calmodulin agarose
CAP‐43: cortical cytoskeleton‐associated protein 23 *alias* BASP1
CHX: cycloheximide
CoIP: co‐immunoprecipitation
ED: effector domain
FOS: Finkel–Biskis–Jenkins murine osteosarcoma oncogene
GST: glutathione *S*‐transferase
HA: hemagglutinin
HEK‐293T: human embryonic kidney 293 cells (large T‐antigen)
hFB: human fibroblasts
IRES: internal ribosomal entry site
JUN: ju‐nana (17) oncogene
KRN1: keratin‐associated protein 1
LUC: luciferase
MARCKS: myristoylated alanine‐rich C‐kinase substrate
MAX: Myc‐associated factor X
MC29: avian myelocytomatosis virus 29
MH2: avian carcinoma virus MH2
MYC: avian myelocytomatosis viral oncogene homolog
Myr‐NT: myristoylated BASP1 amino‐terminal peptide
NK24: avian retrovirus NK24
PKC: protein kinase C
QEF: quail embryo fibroblasts
RAF: rapidly accelerated fibrosarcoma oncogene
RCAS: replication‐competent avian sarcoma leukosis virus vector
RSV: Rous sarcoma virus
SRC: sarc (sarcoma) oncogene
TFP: trifluoperazine
TMX: tamoxifen
TUBA: α‐tubulin
W‐7: *N‐*(6‐aminohexyl)‐5‐chloro‐1‐naphthalenesulfonamide hydrochloride
WT1: Wilms’ tumor 1 protein

## 1 Introduction

The transcription factor MYC constitutes the central hub of a regulatory network controlling the expression of thousands of genes. MYC is a master regulator of fundamental cellular processes such as growth, proliferation, differentiation, metabolism, pluripotency, and apoptosis (Conacci‐Sorrell *et al.*, 2014; Eilers and Eisenman, 2008; Stefan and Bister, 2017). Deregulation of the *MYC* gene by amplification, translocation and enhanced transcriptional activation, or aberrant upstream signaling leads to neoplastic transformation (Dang, 2012; Stefan and Bister, 2017; Stine *et al.*, 2015). Hyperactivation of *MYC* occurs in 60–70% of all human cancers, and *MYC* is classified as a major cancer driver (Dang, 2012; Gabay *et al.*, 2014; Nesbit *et al.*, 1999; Stefan and Bister, 2017; Stine *et al.*, 2015; Tokheim *et al.*, 2016). MYC is a basic helix–loop–helix/leucine zipper protein encompassing protein dimerization domains and a DNA contact surface, forms heterodimers with the Myc‐associated factor X (MAX) protein, and binds to specific DNA sequence elements termed E‐boxes (Conacci‐Sorrell *et al.*, 2014; Eilers and Eisenman, 2008; Nair and Burley, 2003; Stefan and Bister, 2017). In addition to its function as a transcriptional regulator of specific target genes, MYC also acts as a universal amplifier of gene expression controlling broad transcriptional programs (Rahl and Young, 2014; Wolf *et al.*, 2015). Depending on cell and chromatin status, MYC may in fact function along both routes (Dang, 2014; Wolf *et al.*, 2015).

The 25‐kDa brain acid‐soluble protein 1 (BASP1) was originally isolated as a membrane and cytoskeleton‐associated protein from rat and chicken brain (Maekawa *et al.*, 1993; Widmer and Caroni, 1990). It is particularly abundant in nerve terminals during brain development and implicated in neurite outgrowth, maturation of the actin cytoskeleton, and organization of the plasma membrane, but BASP1 is also expressed in various other tissues (Goodfellow *et al.*, 2011; Korshunova *et al.*, 2008). Several cytoplasmic BASP1‐binding proteins have been identified including calmodulin (CaM) (Maekawa *et al.*, 1993; Matsubara *et al.*, 2004; Takasaki *et al.*, 1999) and protein kinase C (PKC) (Maekawa *et al.*, 1993). BASP1 binds to CaM by a small amino‐terminal effector domain (ED) (Maekawa *et al.*, 1993; Matsubara *et al.*, 2004; Takasaki *et al.*, 1999) which is a substrate of PKC and N‐myristoyl transferase. BASP1 belongs to the GAP43/myristoylated alanine‐rich C‐kinase substrate (MARCKS)/CAP‐23 family of myristoylated neuronal growth‐associated proteins and shares distinct biochemical properties with the other members MARCKS and growth‐associated protein 43 which also bind CaM by their basic EDs (Hartl and Schneider, 2019; Mosevitsky, 2005). Phosphorylation by PKC leads to disruption of the interactions of BASP1 with membrane lipids or CaM (Maekawa *et al.*, 1994; Takasaki *et al.*, 1999). BASP1 can then be translocated into the nucleus where it attenuates the transcriptional activity of the Wilms’ tumor suppressor protein WT1, thereby acting as a transcriptional cosuppressor, which also drives cell differentiation processes (Carpenter *et al.*, 2004; Gao *et al.*, 2019; Goodfellow *et al.*, 2011; Toska *et al.*, 2012; Toska *et al.*, 2014).

We have previously reported that transcription of the *BASP1* gene is strongly and specifically repressed in avian cells transformed by the v‐*myc* oncogene (Hartl *et al.*, 2009). Moreover, we showed that ectopic expression of *BASP1* renders fibroblasts resistant to subsequent cell transformation by v‐*myc*, and exogenous delivery of the *BASP1* gene into v‐*myc*‐transformed cells leads to significant attenuation of the transformed phenotype. Based on these discoveries, we proposed that BASP1 displays properties of a putative tumor suppressor (Hartl *et al.*, 2009). BASP1 does not physically interact with v‐Myc (Hartl *et al.*, 2009), but all MYC variant proteins interact with the BASP1‐interaction partner CaM, possibly pointing to a functional connection between these three proteins (Raffeiner *et al.*, 2017). Increased CaM levels indeed enhance the transcriptional and oncogenic activities of v‐Myc (Raffeiner *et al.*, 2017), suggesting that the inhibitory effect of excess BASP1 may be based on interference with the v‐Myc : CaM interaction. Strong support for the proposal that BASP1 acts as a potential tumor suppressor came from recent observations in human and animal cancer. *BASP1* is downregulated in several mammalian tumors including carcinoma, acute and chronic lymphocytic leukemia, and melanoma (Kaehler *et al.*, 2015; Moribe *et al.*, 2008; Ransohoff *et al.*, 2017; Tchernitsa *et al.*, 2004; Wang *et al.*, 2004; Xu *et al.*, 2015; Yeoh *et al.*, 2002). *BASP1* is also downregulated in lung cancer by specific miR‐191‐mediated mRNA degradation (Xu *et al.*, 2015). In mouse, *BASP1* is downregulated among several other anticancer genes in induced cutaneous squamous cell carcinoma by the long noncoding RNA AK144841 (Ponzio *et al.*, 2017). Recently, tumor‐suppressive functions of BASP1 have been observed in several human cancer cell types. Ectopic BASP1 expression inhibits growth of thyroid cancer cell lines and tumor formation in xenografts (Guo *et al.*, 2016). BASP1 binds to the estrogen receptor‐α and acts as a transcriptional corepressor thus enhancing the effect of the estrogen antagonist tamoxifen (TMX) (Marsh *et al.*, 2017). BASP1 elicits tumor suppressor activity in breast cancer, and BASP1 expression levels correlate with increased patient survival (Marsh *et al.*, 2017). Methylation‐associated silencing of *BASP1* contributes to leukemogenesis in acute myeloid leukemia (AML). Ectopic BASP1 expression inhibits proliferation and colony formation of AML cell lines by inducing apoptosis and cell cycle arrest (Zhou *et al.*, 2018). The antitumor isoflavonoid genistein increases BASP1 expression in human prostate cancer (Zhang *et al.*, 2019). In pancreatic cancer, expression of *BASP1* prolongs survival whereas tumors with no *BASP1* but high *WT1* expression indicate a poor prognosis (Zhou *et al.*, 2019).

In this report, we confirm and extend the analysis of specific MYC : CaM binding and show that excess BASP1 or a synthetic BASP1 ED peptide displaces v‐Myc from CaM leading to enhanced v‐Myc proteolysis. Moreover, the inhibitory effects of BASP1 on v‐Myc‐mediated transcriptional activation or cell transformation are partially compensated by ectopic CaM, suggesting that BASP1 inhibits v‐Myc by sequestration from CaM. In view of the tumor‐suppressive potential of BASP1 in human cancer, small compounds or peptides based on the BASP1 ED structure could be developed to expand the spectrum of therapeutic approaches for the treatment of cancers with high *MYC* expression.

## 2 Methods

### 2.1 Cell culture and retroviruses

Primary quail embryo fibroblasts (QEF) and QEF transformed by the v‐*myc* (QEF/RCAS‐MC29), v‐*fos* (QEF/NK24), v‐*jun* (QEF/ASV17), v‐src (QEF/RSV), or v‐*myc*/v‐*mil* (QEF/MH2) oncogenes were generated by infection with the corresponding retroviruses and grown as described (Hartl *et al.*, 2009). Quantification of cell transformation by focus or colony formation was performed as described (Hartl *et al.*, 2009; Raffeiner *et al.*, 2017). QEF transfected with the replication‐competent pRCAS vector (QEF/RCAS) or with the pRCAS derivatives pRCAS‐MC29, pRCAS‐BASP1, and pRCAS‐MC29‐IRES‐BASP1 have been described (Hartl *et al.*, 2009). The constructs pRCAS‐BASP1(G2A), pRCAS‐BASP1(K7‐10A), pRCAS‐BASP1(K4A, L5A), and pRCAS‐BASP1(S6A) have been generated by *in vitro* mutagenesis as described (Hartl *et al.*, 2009). To construct pRCAS‐CALM1‐IRES‐BASP1, the coding region of the chicken calmodulin gene (*CALM1*) was first inserted into pRCAS to generate pRCAS‐CALM1. Then, a segment containing the internal ribosomal entry site IRES‐BASP1 portion was ligated into pRCAS‐CALM1 to yield pRCAS‐CALM1‐IRES‐BASP1. The nonproducer cell line QEF/Rc‐myc expressing the v‐*myc* allele from MC29 has been described (Hartl *et al.*, 2009). QT6 cells are a line of chemically transformed QEF (Moscovici *et al.*, 1977) with c‐*myc* expression levels comparable to those in normal QEF (Reiter *et al.*, 2007). Calcium phosphate‐mediated transfection or nucleofection of DNA into fibroblasts was done as described (Hartl *et al.*, 2009; Hartl *et al.*, 2001). Cultivation of human immortalized skin fibroblasts (hFB) or epithelial kidney cells (HEK‐293T), and of the human cancer cell lines K‐562, MOLT‐4, and SW‐480, which are derived from chronic myelogenous leukemia, acute lymphoblastic leukemia, and colorectal adenocarcinoma, respectively, has been described (Raffeiner *et al.*, 2014; Valovka *et al.*, 2013).

### 2.2 Expression plasmids, gene transfer, reporter gene assay, and cell proliferation analysis

The expression plasmid pcDNA3.1‐HA‐c‐MYC contains the hemagglutinin (HA)‐tagged coding sequence of the human *MYC* gene inserted into the pcDNA3.1 vector (Raffeiner *et al.*, 2017). The pRc/RSV‐derived eukaryotic expression vectors pRc‐HA‐v‐Myc, pRc‐v‐Myc, pRc‐v‐Fos, pRc‐v‐Src, pRc‐BASP1, and pRc‐CALM1 have been described (Hartl *et al.*, 2009; Hartl *et al.*, 2001; Raffeiner *et al.*, 2017). For construction of DNA templates encoding amino‐terminally FLAG‐tagged proteins, a double‐stranded oligodeoxynucleotide encoding the nine‐amino acid peptide tag DYKDDDDKD was inserted between codons 1 and 2 of chicken *CALM1* or of human keratin‐associated protein 5.9 (*KRN1*) to generate pRc‐FLAG‐CALM1 and pRc‐FLAG‐KRN1 as described (Hartl *et al.*, 2001). DNA transfection or nucleofection was performed as described (Hartl *et al.*, 2009; Raffeiner *et al.*, 2017). Transcriptional transactivation analysis using the luciferase (LUC) reporter system including the LUC constructs pGL3‐Basic (pLUC) and pGL3‐WS5 (pLUC‐WS5) has been described (Raffeiner *et al.*, 2017; Valovka *et al.*, 2013). Proliferation of cells treated with trifluoperazine (TFP) or the N‐terminal BASP1 ED peptide (Myr‐NT) was monitored in real time by using the live‐cell imaging system IncuCyte S3 (Essen Bioscience/Sartorius, Vienna, Austria). Cells were seeded in a 96‐well dish (Corning, Vienna, Austria) and incubated overnight. TFP or Myr‐NT was then added to final concentrations of 5–20 or 40–80 µm, respectively. Cells were monitored for 3 days by phase‐contrast imaging every 8 h from four separate regions per well using a 10× objective.

### 2.3 Chemicals, peptides, and antibodies

The calmodulin inhibitor TFP (Merck, Vienna, Austria) was dissolved in H_2_O at 50 mm, the protein translation inhibitor cycloheximide (CHX; Sigma‐Aldrich, Vienna, Austria) in ethanol at 100 mm, and the proteasome inhibitor MG‐132 (Axon Medchem, Groningen, the Netherlands) in DMSO at 10 mm. The peptides Myr‐NT (myristoyl‐GGKLSKKKKG‐OH) and Myr‐CT (myristoyl‐GSDQTIAVQD‐OH) corresponding to the chicken BASP1 amino or carboxyl terminus, respectively (Hartl *et al.*, 2009), and the control peptides Myr‐FL (myristoyl‐GDYKDDDDKD) or NT (MGGKLSKKKKGYNVNC) were commercially synthesized (PANATecs, Heilbronn, Germany; Biotrend, Cologne, Germany). Myr‐NT, Myr‐FL, and NT were dissolved in H_2_O at 10 mm and Myr‐CT in 75% DMSO at 2.5 mm. The peptide B‐CT (H_2_N‐SEAPATNSDQTIAVQ‐OH) corresponding to amino acid residues 229–243 of chicken BASP1 was dissolved in H_2_O at 1 mm (Hartl *et al.*, 2009). Specific rabbit antisera recognizing v‐Myc (anti‐Myc‐CT, anti‐Myc‐NT), v‐Fos, BASP1, or Max have been described (Hartl *et al.*, 2010; Hartl *et al.*, 2009; Hartl *et al.*, 2001; Reiter *et al.*, 2007; Valovka *et al.*, 2013). Mouse antibodies directed against α‐tubulin, CaM, Src, and the HA or FLAG tags have been described (Hartl *et al.*, 2001; Raffeiner *et al.*, 2017).

### 2.4 Protein analyses

SDS/PAGE, immunoblotting, *in vitro* translation, and immunoprecipitation were carried out as described (Hartl *et al.*, 2009; Hartl *et al.*, 2001; Raffeiner *et al.*, 2017). The construct pBS‐CALM1 was created by inserting the coding region of the chicken calmodulin 1 (*CALM1*) gene into the Bluescript vector II SK (+) (Hartl *et al.*, 2006; Raffeiner *et al.*, 2017). Protein pull‐down assays using calmodulin agarose (CaM‐ag.) beads or a glutathione *S*‐transferase (GST)/calmodulin fusion protein coupled to glutathione Sepharose beads were done as described (Raffeiner *et al.*, 2017). Co‐immunoprecipitation (CoIP) analysis was carried out by cell lysis and precipitation with the first antibody under native conditions as described (Hartl *et al.*, 2009; Reiter *et al.*, 2007). The subsequent precipitation was performed using the second antibody under denaturing conditions (Hartl *et al.*, 2009; Reiter *et al.*, 2007) followed by SDS/PAGE and immunoblotting, again using the second antibody. For densitometry, relative protein levels were determined with ImageQuant TL (GE Healthcare, Vienna, Austria) as described (Raffeiner *et al.*, 2017).

## 3 Results

### 3.1 Specificity of MYC : CaM interaction and transformation inhibition by BASP1

**Figure 1:**
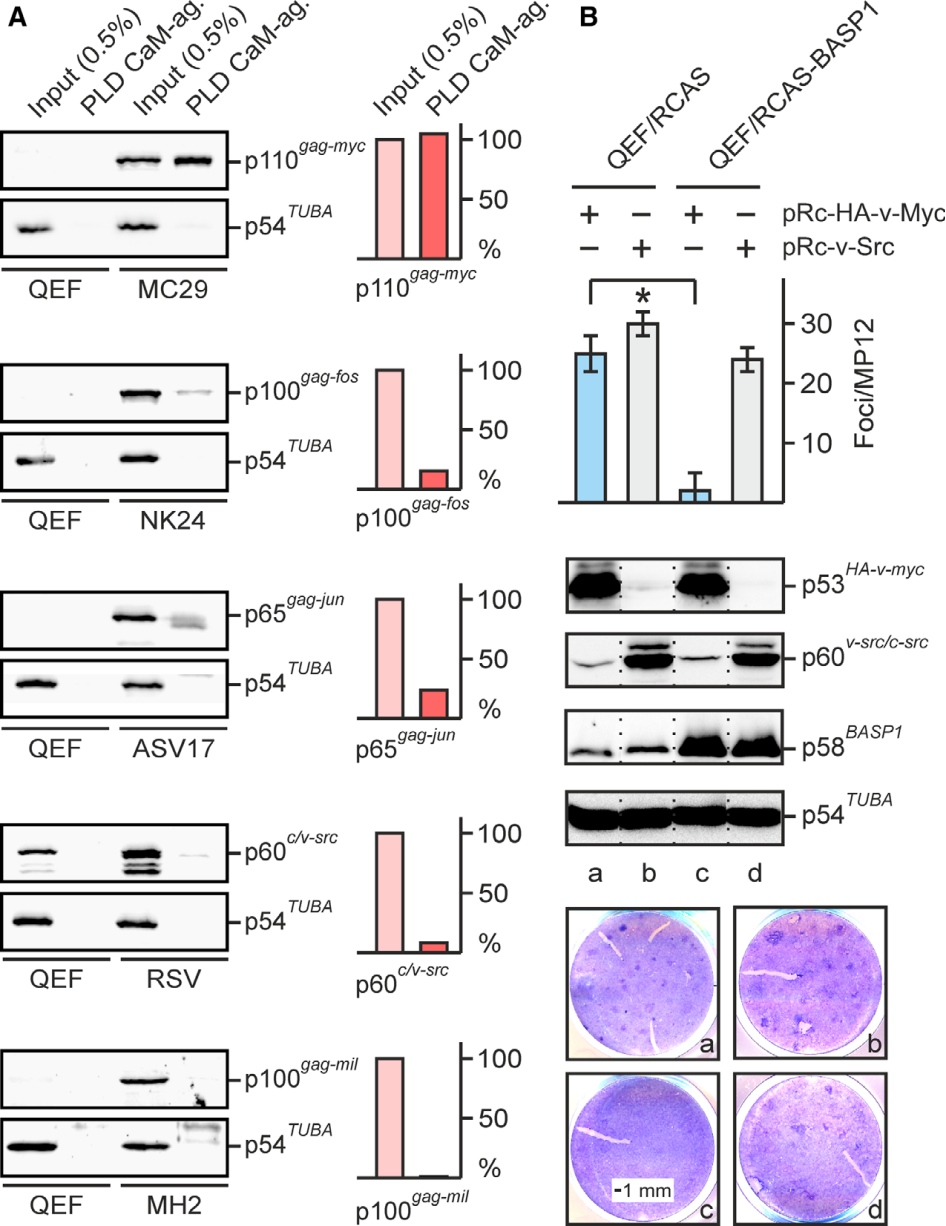
Specific binding of CaM to v‐Myc, and specific inhibition of v‐Myc‐induced cell transformation by the CaM‐binding protein BASP1. (A) Pull‐down assay using CaM‐ag. as affinity matrix and cell lysates prepared from normal QEF and from QEF/MC29, QEF/NK24, QEF/ASV17, QEF/RSV, and QEF/MH2 transformed by the MC29, NK24, ASV17, RSV, and MH2 retroviruses, expressing the p110 Gag‐Myc, p100 Gag‐Fos, p65 Gag‐Jun, p60 v‐Src, and p100 Gag‐Mil oncoproteins, respectively. Left panel: Proteins eluted from the CaM‐agarose beads were subjected to SDS/PAGE and immunoblotting (*n* = 2) using antibodies directed against the corresponding viral oncoproteins or α‐tubulin. The protein reacting with anti‐Src in the QEF input lane is endogenous c‐Src. Right panel: The relative quantifications of input (pink) and pull‐down (red) signals from QEF/MC29, QEF/NK24, QEF/ASV17, QEF/RSV, and QEF/MH2 are shown next to the blots. (B) Specific inhibition of v‐Myc‐induced cell transformation by BASP1 overexpression. QEF were transfected with pRCAS‐BASP1 or with the empty pRCAS vector, passaged four times (QEF/RCAS, QEF/BASP1), and then supertransfected with the eukaryotic expression vectors pRc‐HA‐v‐Myc or pRc‐v‐Src encoding the v‐*myc* (v‐*myc* allele without *gag*) or v‐*src* oncogenes, respectively. Cells were kept under agar overlay for 21 days and then stained with eosin methylene blue (lower panel). Foci were counted on MP12 dishes (*n* = 2). Vertical bars show standard deviations (SD) from triplicates (upper panel). Statistical significance was assessed by using a paired Student’s *t*‐test (*P < 0.05). Proteins were analyzed by immunoblotting (middle panel) using equal amounts of cell extracts prepared 1 day after supertransfection and specific antisera directed against Myc, Src, BASP1, or α‐tubulin (*n* = 2). The dotted lines mark splicing sites in the blot images, from which three redundant lanes have been removed.

We have previously shown that v‐Myc does not physically interact with BASP1 (Hartl *et al.*, 2009) but with the BASP1‐binding partner CaM in a calcium‐dependent manner (Raffeiner *et al.*, 2017). On the other hand, BASP1 specifically inhibits v‐Myc‐induced cell transformation and transcriptional activation (Hartl *et al.*, 2009). To investigate whether there is a link between the strong v‐Myc binding to CaM and the specific transformation inhibition by BASP1, the specificity of the v‐Myc : CaM interaction was assessed in a protein pull‐down assay. Cell extracts were prepared from QEF and from QEF transformed by the v‐*myc*, v‐*fos*, v‐*jun*, v‐*src*, or v‐*mil*/v‐*myc* oncogenes. The extracts were incubated with CaM cross‐linked to agarose. CaM‐binding proteins were then specifically detected using antibodies directed against the v‐Myc, v‐Fos, v‐Jun, v‐Src, or v‐Mil oncoproteins. The untransformed QEF were used as a negative control (Fig. 1A). Strong binding between v‐Myc and CaM was observed, whereas only weak interactions were detected for the transcription factors v‐Fos and v‐Jun, and no binding for the serine/threonine kinase v‐Mil (Raf), demonstrating the strength and specificity of the previously reported v‐Myc : CaM interaction (Raffeiner *et al.*, 2017). In addition, weak binding to the cytoplasmatic tyrosine kinase v‐Src was observed (Fig. 1A) in agreement with recent results that Src interacts with CaM, both in the calcium‐bound and in the apo form (Stateva *et al.*, 2015).

To analyze the specificity of transformation inhibition by BASP1, QEF were first transfected with the retroviral vector RCAS‐BASP1 or the empty RCAS vector and then supertransfected with expression vectors encoding v‐Myc or v‐Src proteins (Fig. 1B). Whereas v‐Src efficiently transforms QEF independent of endogenous or ectopic BASP1 protein levels, v‐Myc‐induced cell transformation is strongly inhibited by ectopic BASP1 expression (Fig. 1B), in agreement with previous results (Hartl *et al.*, 2009). To test whether distinct N‐terminal BASP1 residues mediating CaM binding (Matsubara *et al.*, 2004) are required for inhibition of v‐Myc‐induced cell transformation, a mutational analysis of the BASP1 N terminus was carried out (Fig. S1). Residues implicated in CaM binding include the myristoylation site (G2) with a conjugated myristoyl moiety, the nuclear localization signal (K7‐10), and the lysine (K4) and leucine (L5) residues within the PKC phosphorylation signal (KLS; Fig. S1A). To analyze transformation inhibition by BASP1, QEF were first transfected with the empty RCAS vector or with wild‐type or mutant retroviral RCAS‐BASP1 vectors and then supertransfected with an expression vector encoding v‐Myc (Fig. S1B). Whereas the G2A and K7‐10A mutations efficiently abolish the inhibitory BASP1 function as reported recently (Hartl *et al.*, 2009), the K4A/L5A mutant retains a partial capacity to inhibit v‐Myc‐induced cell transformation. Mutation of the adjacent serine residue (S6) had no effect on the inhibitory BASP1 function indicating that serine‐6 phosphorylation is not required (Fig. S1C). In fact, serine 6‐phosphorylation even impedes the interaction with CaM (Maekawa *et al.*, 1994). The capacity of the mutant BASP1 proteins to interact with CaM was evaluated by a protein pull‐down assay using the relevant cell extracts and CaM fused to the GST protein (Fig. S1D). The result shows that under these *in vitro* conditions, only BASP1 and the S6A mutant, which completely inhibit v‐Myc‐induced cell transformation (Fig. S1C), are able to efficiently bind to glutathione Sepharose‐immobilized CaM confirming the structural data (Matsubara *et al.*, 2004). The K4A/L5A mutant, which partially inhibits focus formation, only retains a marginal capacity to bind to CaM. The mutational analysis suggests that the BASP1 : CaM interaction is implicated or at least contributes in inhibition of v‐Myc‐induced cell transformation.

### 3.2 The MYC : CaM interaction is perturbed in the presence of ectopic BASP1

**Figure 2:**
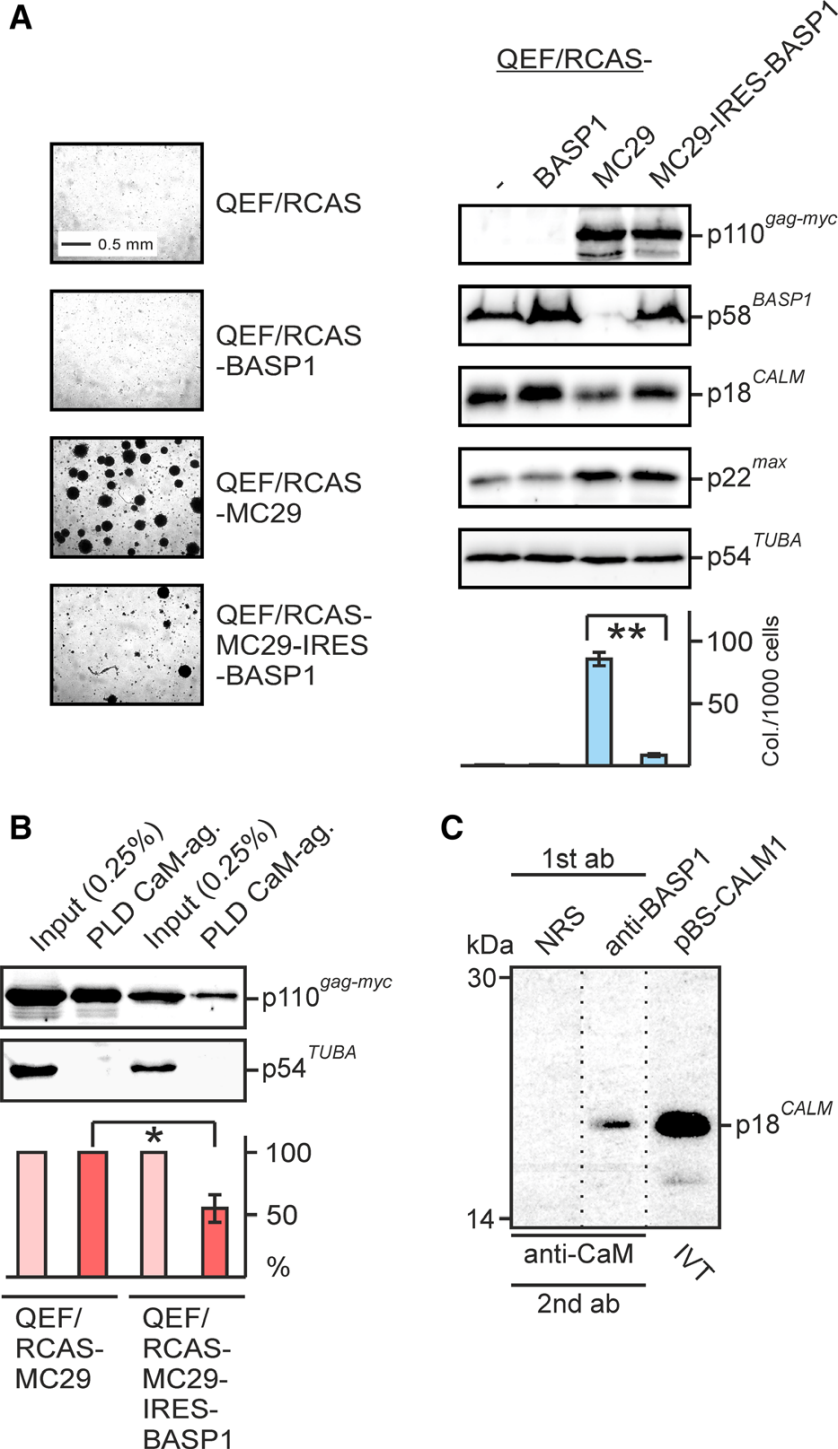
Impaired v‐Myc : CaM interaction in cells co‐expressing v‐Myc and BASP1. (A) Suppression of the transformed phenotype of cells co‐expressing v‐Myc (Gag‐Myc) and BASP1 encoded by the bicistronic pRCAS‐MC29‐IRES‐BASP1 vector. Equal numbers of cells (5 × 10^3^) transfected with pRCAS‐BASP1, pRCAS‐MC29 encoding the original Gag‐Myc fusion protein, pRCAS‐MC29‐IRES‐BASP1, or the empty pRCAS vector were seeded in soft agar on MP24 dishes and incubated for 12 days. Bright‐field micrographs of agar colonies and a quantification of colonies per 1000 cells seeded are shown (*n* = 2; left panel). Standard deviations (SD) from triplicates are shown by vertical bars. Statistical significance was assessed by using a paired Student’s *t*‐test (**P < 0.01; lower right panel). Proteins were analyzed by immunoblotting (*n* = 2) using equal amounts of cell extracts (2.5 × 10^5^ cells) and specific antisera directed against v‐Myc, BASP1, CaM, Max, and α‐tubulin (upper right panel). (B) Pull‐down assay using CaM‐agarose as affinity matrix and lysates prepared from QEF/RCAS‐MC29 and QEF/RCAS‐MC29‐IRES‐BASP1 cells expressing the Gag‐Myc or the Gag‐Myc plus BASP1 proteins, respectively. Proteins eluted from the CaM‐agarose beads were subjected to SDS/PAGE and immunoblotting using antibodies directed against v‐Myc, α‐tubulin, or BASP1. Relative quantifications of input (pink) and pull‐down (red) signals (*n* = 3) are shown below. The standard deviation (SD) is shown by a vertical bar. Statistical significance of the reduced CaM binding in cells co‐expressing BASP1 was assessed by using a paired Student’s *t*‐test (*P < 0.05). (C) BASP1 : CaM interaction determined by CoIP analysis. Aliquots of a metabolically [^35^S]‐methionine‐labeled QEF extract prepared under native conditions were incubated with normal rabbit serum (NRS) or polyclonal antibodies directed against the BASP1 protein (anti‐BASP1). Precipitated proteins were dissociated and subsequently immunoprecipitated under denaturing conditions using anti‐CaM as the second antibody. Immunoprecipitates were analyzed by SDS/PAGE and fluorography together with *in vitro* translated (IVT) CaM encoded by a Bluescript vector (pBS‐CALM1). The dotted lines mark splicing sites in the fluorographs, from which two redundant lanes have been removed.

To test whether ectopic *BASP1* expression interferes with the v‐Myc : CaM interaction, QEF were transfected with the retroviral pRCAS‐MC29 vector containing the v‐*myc* oncogene or with the bicistronic pRCAS‐MC29‐IRES‐BASP1 construct containing v‐*myc* and *BASP1* genes (Hartl *et al.*, 2009). Both cell types efficiently express the Gag‐Myc hybrid protein. Control cells were transfected with the empty pRCAS vector or with pRCAS‐BASP1 encoding the *BASP1* gene only (Fig. 2A). Endogenous BASP1 is expressed in normal QEF transfected by the control RCAS vector and specifically suppressed in QEF/RCAS‐MC29 cells, as reported previously (Hartl *et al.*, 2009). Ectopic BASP1 is expressed in QEF/RCAS‐BASP1 and QEF/RCAS‐MC29‐IRES‐BASP1 cells, whereas CaM and the MYC dimerization partner MAX are expressed in all cell types although we constantly observe slightly increased or decreased CaM levels in cells ectopically expressing BASP1 or MYC, respectively. Furthermore, there are enhanced levels of the dimerization partner MAX in cells overexpressing MYC (Fig. 2A). QEF/RCAS‐MC29 cells are highly transformed leading to efficient colony formation in soft agar, whereas cells co‐expressing v‐Myc and BASP1 display a drastically reduced transformed phenotype (Fig. 2A). No colony formation was observed for QEF/RCAS and QEF/RCAS‐BASP1 control cells. To measure the efficiency of the v‐Myc : CaM interaction in cells co‐expressing v‐Myc and BASP1, a protein pull‐down assay was performed using CaM‐agarose and cell extracts derived from QEF/RCAS‐MC29 and QEF/RCAS‐MC29‐IRES‐BASP1. The amount of CaM‐bound v‐Myc relative to input levels was reduced to about 50% in cells containing high v‐Myc and BASP1 levels, as compared to cells expressing v‐Myc only (Fig. 2B). The established BASP1 : CaM interaction (Maekawa *et al.*, 1993; Matsubara *et al.*, 2004; Takasaki *et al.*, 1999) was confirmed by CoIP analysis (Fig. 2C), in addition to the above‐described GST‐CaM protein pull‐downs (cf. Fig. S1D).

**Figure 3:**
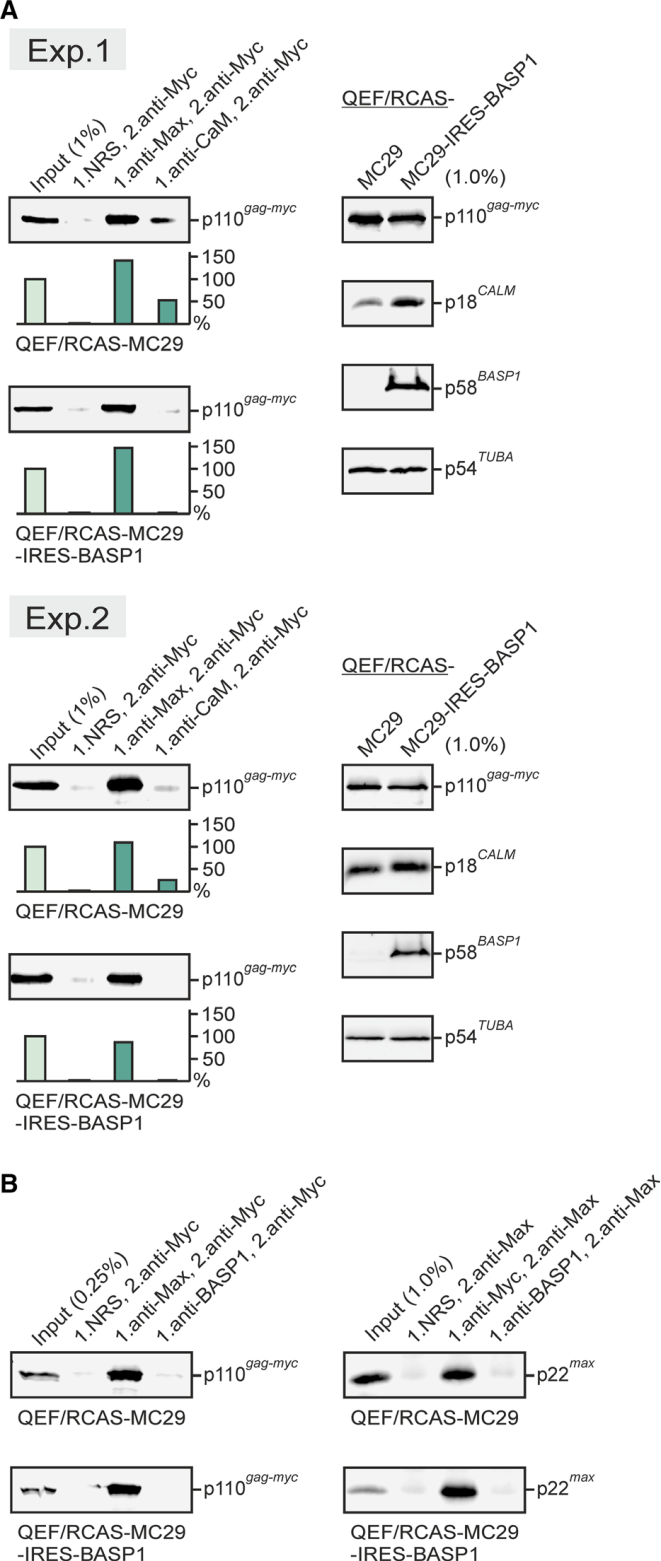
Ectopic BASP1 expression correlates with an impaired v‐Myc : CaM interaction. (A) Left panel: CoIP analysis from two independent experiments (Exp. 1/2) using extracts from QEF/RCAS‐MC29 and QEF/RCAS‐MC29‐IRES‐BASP1 cells and Max‐ or CaM‐specific antibodies (anti‐Max, anti‐CaM) for the first precipitation under native conditions and a v‐Myc‐specific antibody (anti‐Myc) for the second precipitation under denaturing conditions. Normal rabbit serum (NRS) and detection of the v‐Myc : Max interaction were used as negative and positive controls, respectively. Precipitated proteins were dissociated and analyzed by SDS/PAGE and immunoblotting using anti‐Myc. Relative quantifications of input (light green) and CoIP signals (green) are shown below the blots. Right panel: Immunoblotting of input samples using v‐Myc‐, CaM‐, BASP1‐, and α‐tubulin‐specific antibodies. (B) CoIP analysis as described in (A) using anti‐Max, anti‐Myc, or anti‐BASP1 as first antibodies, and anti‐Myc or anti‐Max as second antibodies, respectively. Immunoblots were performed using anti‐Myc or anti‐Max, respectively. No direct interactions between BASP1 and v‐Myc or between BASP1 and Max were detectable in agreement with previous observations (Hartl *et al.*, 2009; Raffeiner *et al.*, 2017).

The interference of the BASP1 protein with the v‐Myc : CaM interaction was also tested *in vivo* by CoIP analysis. Cell extracts were prepared under native conditions from QEF/RCAS‐MC29 and QEF/RCAS‐MC29‐IRES‐BASP1 cells, and protein precipitation was performed first with antibodies directed against MAX or CaM, or with normal rabbit serum. Precipitation under denaturing conditions with a second antibody directed against v‐Myc confirmed that in both cell types, v‐Myc efficiently interacts with its dimerization partner MAX (Fig. 3A). Furthermore, there is a v‐Myc : CaM interaction in QEF/RCAS‐MC29 cells expressing v‐Myc, but not in QEF/RCAS‐MC29‐IRES‐BASP1 cells containing v‐Myc and ectopic BASP1. Apparently, the presence of BASP1 impedes the v‐Myc : CaM interaction despite equal v‐Myc and even elevated CaM levels in QEF/RCAS‐MC29‐IRES‐BASP1 cells (Fig. 3A). This assay was also used to confirm that there are no direct interactions between BASP1 and v‐Myc or MAX (Hartl *et al.*, 2009) (Fig. 3B). The results from protein pull‐down and CoIP analyses suggest that BASP1 competes with v‐Myc for CaM binding.

### 3.3 Reduced stability of the MYC protein in cells with ectopic BASP1 expression

**Figure 4:**
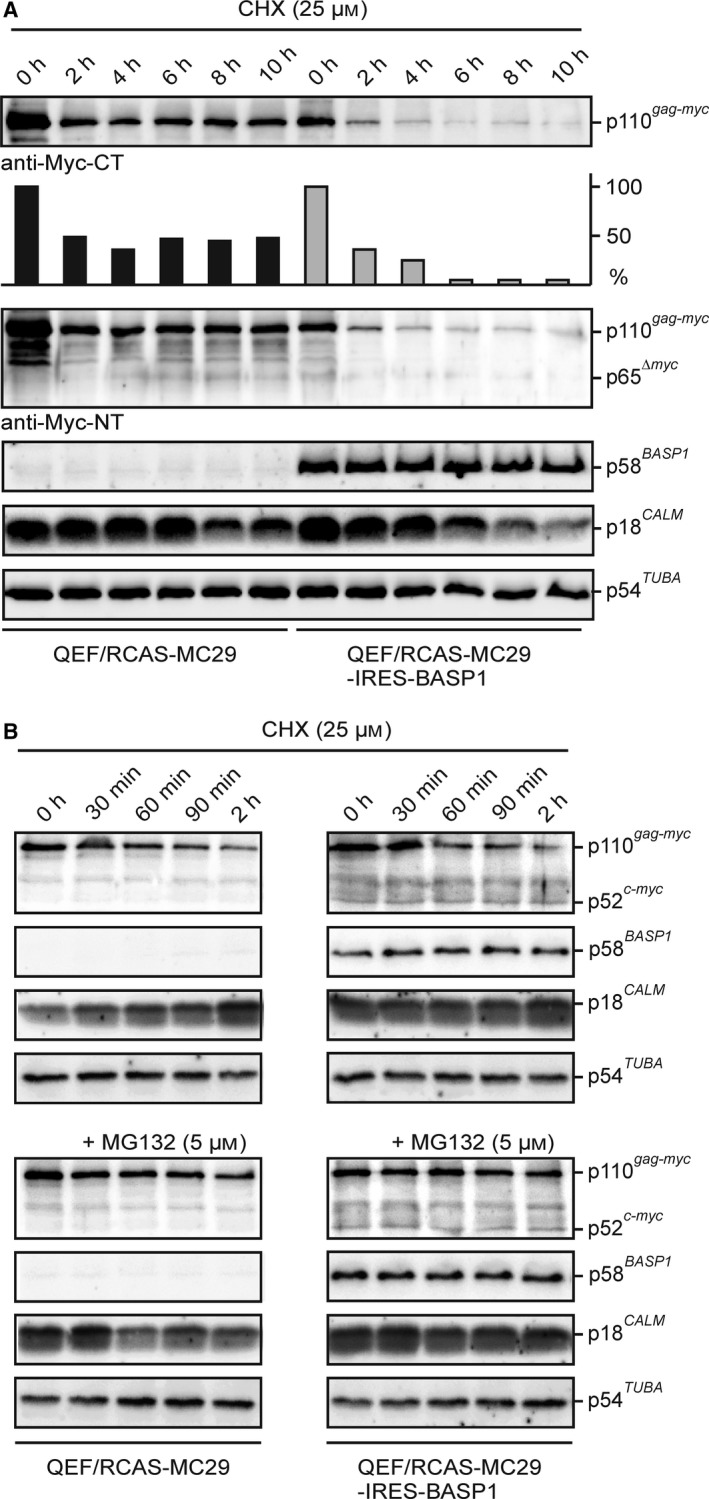
Ectopic BASP1 expression correlates with decreased Gag‐Myc protein stability. (A) Gag‐Myc protein stability in the presence of ectopic BASP1. Equal numbers (2.5 × 10^5^) of QEF/RCAS‐MC29 and QEF/RCAS‐MC29‐IRES‐BASP1 cells were seeded onto MP6 wells and incubated in the presence of 25 µm CHX. Cell extracts were prepared after the indicated time points (0–10 h). Proteins were analyzed by immunoblotting using equal amounts of cell extracts and specific antisera directed against the carboxyl terminus (CT) or amino terminus (NT) of v‐Myc, BASP1, CaM, or α‐tubulin. A specific degradation product retaining the v‐Myc N terminus with an apparent molecular mass of 65 kDa was detected. A representative experiment (*n* = 2) is shown. A quantification of the v‐Myc protein expression levels is depicted below the upper MYC blot. (B) Kinetics of the initial phase (0–2 h) of protein translation inhibition as shown under (A). Equal numbers of QEF/RCAS‐MC29 (1.0 × 10^5^) and QEF/RCAS‐MC29‐IRES‐BASP1 (1.5 × 10^5^) cells were seeded onto MP12 wells and incubated in the presence of 25 µm CHX, and in the presence or absence of the proteasome inhibitor MG‐132 (5 µm). Cell extracts were prepared after the indicated time points. Proteins were analyzed by immunoblotting using equal amounts of cell extracts and specific antisera directed against v‐Myc/c‐Myc, BASP1, CaM, or α‐tubulin.

We have shown recently that overexpressed CaM increases the transcriptional activation and cell transformation potential of v‐Myc (Raffeiner *et al.*, 2017). To test whether the blocked v‐Myc : CaM interaction in QEF/RCAS‐MC29‐IRES‐BASP1 cells affects v‐Myc protein stability, cells were incubated in the presence of the protein translation inhibitor CHX. In QEF/RCAS‐MC29 cells, the endogenous BASP1 protein is downregulated due to transcriptional suppression of the *BASP1* gene by v‐Myc (Hartl *et al.*, 2009). A time course of up to 10 h was performed showing that after 2 h, v‐Myc protein levels decrease in both cell types to about 50% (Fig. 4A). Whereas in QEF/RCAS‐MC29 cells v‐Myc levels then remain constant during the entire time course, in QEF/RCAS‐MC29‐IRES‐BASP1 cells the v‐Myc protein is significantly less stable and almost completely degraded after 4 h. In addition, a reduction in CaM levels was observed after 6 h in these cells. In contrast, ectopic BASP1 expression remains remarkably stable after 10 h, a time point where even the stability of α‐tubulin slightly decreases (Fig. 4A). To test whether the observed MYC and CaM degradation depend on the ubiquitin/proteasome pathway (Farrell and Sears, 2014), more detailed kinetics were performed in the presence of the proteasome inhibitor MG‐132 (Fig. 4B, Fig. S2). In both cell types, MG‐132 led to v‐Myc and CaM stabilization, suggesting that both proteins are degraded according to the same pathway. In contrast to v‐Myc, endogenous c‐Myc, only detectable in QEF/RCAS‐MC29‐IRES‐BASP1 but not in cells transformed by v‐Myc (Hartl and Bister, 1998; Penn *et al.*, 1990), remained stable during the first 2 h of the time course (Fig. 4B). These results suggest that inhibition of v‐Myc‐induced cell transformation by BASP1 could be caused by decreased v‐Myc protein stability, which is probably mediated by the ubiquitin/proteasome pathway. To analyze the functional relevance of CaM in this context, the CaM inhibitor TFP (Vandonselaar *et al.*, 1994) was added to cells whose protein synthesis has been blocked by CHX (Fig. S2). In the presence of TFP, the amounts of v‐Myc were slightly reduced in the absence and in the presence of BASP1, indicating that CaM inhibition contributes to enhanced v‐Myc degradation. On the other hand, CaM levels slightly increase in QEF/RCAS‐MC29 after 6‐h CHX and TFP treatment, whereas in QEF/RCAS‐MC29‐IRES‐BASP1 cells, an opposite effect was observed (Fig. S2).

### 3.4 The BASP1 effector domain acts like a CaM inhibitor and interferes with MYC‐dependent oncogenesis

**Figure 5:**
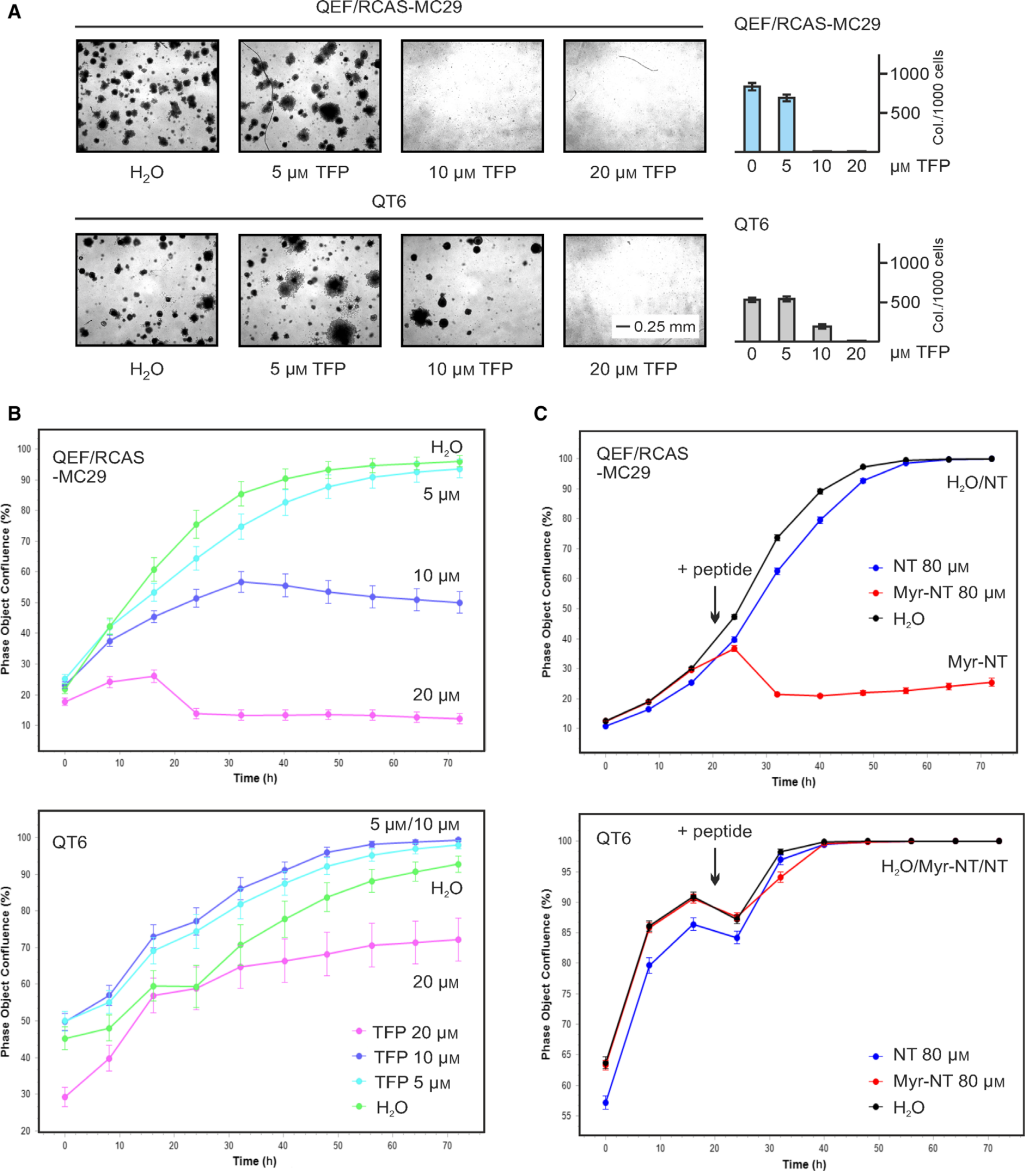
Pharmacological CaM inhibition in v‐Myc‐transformed cells. (A) Equal numbers (5 × 10^3^) of QEF/RCAS‐MC29 cells encoding the original Gag‐Myc fusion protein or chemically transformed QT6 cells were seeded in soft agar onto MP24 dishes in the presence of increasing concentrations of the CaM inhibitor TFP and incubated for 17 days. Bright‐field micrographs of agar colonies (left panel) and a quantification of colonies per 1000 cells seeded are shown (right panel). Standard errors of the mean (SEM) from independent experiments (*n* = 2) done in triplicate are shown by vertical bars. Statistical significance was assessed by using a paired Student’s *t*‐test (*P < 0.05). (B, C) Proliferation inhibition of QEF/RCAS‐MC29 cells by the CaM inhibitor TFP (B) or by a myristoylated BASP1 ED peptide (Myr‐NT) (C). QEF/RCAS‐MC29 cells (5 × 10^3^) or, as a control, chemically transformed QT6 cells (2 × 10^4^) were seeded onto 96‐well cell culture plates. The next day, TFP, Myr‐NT, or the nonmyristoylated control peptide NT was added at the indicated final concentrations and cell densities measured every 8 h over a 3‐day time period using an IncuCyte live‐cell analysis system. Cells without treatment (H_2_O) were used as reference.

To analyze the effect of CaM inhibition on cell transformation, TFP was added to v‐*myc*‐transformed QEF/RCAS‐MC29 cells, and anchorage‐independent growth was monitored by colony formation in semisolid medium (Fig. 5A). TFP efficiently inhibits colony formation of v‐Myc‐transformed cells at a 10 µm concentration in contrast to the chemically transformed QT6 control cell line (Moscovici *et al.*, 1977), where a higher TFP concentration of 20 µm was required to block soft agar colony formation (Fig. 5A). To test for a direct effect on proliferation, cell growth of QEF/RCAS‐MC29 was monitored for 3 days upon TFP addition to the culture medium. As a control, QT6 cells were incubated under the same conditions (Fig. 5B). At 10 µm TFP concentration, the proliferation rate of QEF/RCAS‐MC29 cells was reduced to about 50% and almost abolished at 20 µm. In contrast, the proliferation of QT6 cells was not affected at 10 µm and only reduced to 70% at 20 µm TFP. Because the BASP1 ED with intact myristoylation signal binds to CaM and suffices to inhibit v‐Myc‐induced cell proliferation (Hartl *et al.*, 2009), a myristoylated N‐terminal BASP1 peptide (Myr‐NT) was added to the culture medium of QEF/RCAS‐MC29 and QT6 cells (Fig. 5C). A nonmyristoylated derivative (NT) was used as a control. Like TFP, Myr‐NT specifically inhibited the proliferation of v‐Myc‐transformed cells, suggesting that this BASP1 protein domain may act as a CaM inhibitor although high peptide concentrations (40–80 µm) were required to see a specific effect (Fig. S3A). However, we point out that upon transfection of Myr‐NT, concentrations in the low micromolar range are sufficient to obtain a biological effect (see below). To test whether the TFP‐ or Myr‐NT‐mediated inhibition of QEF/RCAS‐MC29 proliferation correlates with an impaired binding of v‐Myc to CaM, a protein pull‐down was performed in the absence or presence of TFP or Myr‐NT using [^35^S]‐pulse‐labeled cell extracts. TFP reduced the Ca^2+^‐dependent binding of v‐Myc to CaM‐agarose to about 50% (Fig. S3B). Due to the excess of matrix‐bound CaM, the application of higher TFP concentrations (40 µm) was necessary. A significant reduction in CaM binding was also caused by the Myr‐NT peptide at the same concentration (40 µm), although slight reductions of v‐Myc binding to immobilized CaM were also observed in the presence of two unrelated control peptides (Myr‐CT, B‐CT; Fig. S3B). Having identified the myristoylated N‐terminal BASP1 peptide as a reagent to selectively interfere with the viability of v‐*myc*‐transformed cells, we explored if Myr‐NT would also inhibit the growth of leukemia cells containing high levels of endogenous *MYC* (Nesbit *et al.*, 1999; Valovka *et al.*, 2013) (Fig. S4A). The amino acid sequence encompassed by Myr‐NT is 100% identical among chicken, human, and other species (Hartl *et al.*, 2009). Again, for efficient cellular uptake, high concentrations of Myr‐NT (80 µm) were required when the peptide was added to the cells without transfection agent. Specific proliferation inhibition of the nonadherent leukemia cell lines K‐562, MOLT‐4 was observed, and also to some extent in the colon carcinoma cell line SW‐480, which grows adherently (Fig. S4A). Normal human fibroblasts (hFB) and the epithelial kidney cell line HEK‐293T served as controls. In contrast, a myristoylated control peptide (Myr‐FL) did not interfere with cell growth showing that myristoylation *per se* has no toxic effect to the cells. Only this post‐translational modification in combination with the highly conserved residues 2–11 from BASP1 must account for the observed cell‐killing effect. Expression analysis of the endogenous *MYC*, *BASP1*, and calmodulin (*CALM1‐3*) genes revealed that cells with high amounts of *MYC* and low amounts of *BASP1* and *CALM1‐3* displayed the highest susceptibility toward the Myr‐NT peptide (Fig. S4B). Hence, this result suggests that BASP1‐mediated inhibition depends on actual MYC and CaM levels in human cancer cells.

### 3.5 BASP1‐mediated inhibition of transcriptional activation by MYC is compensated by ectopic CaM expression

**Figure 6:**
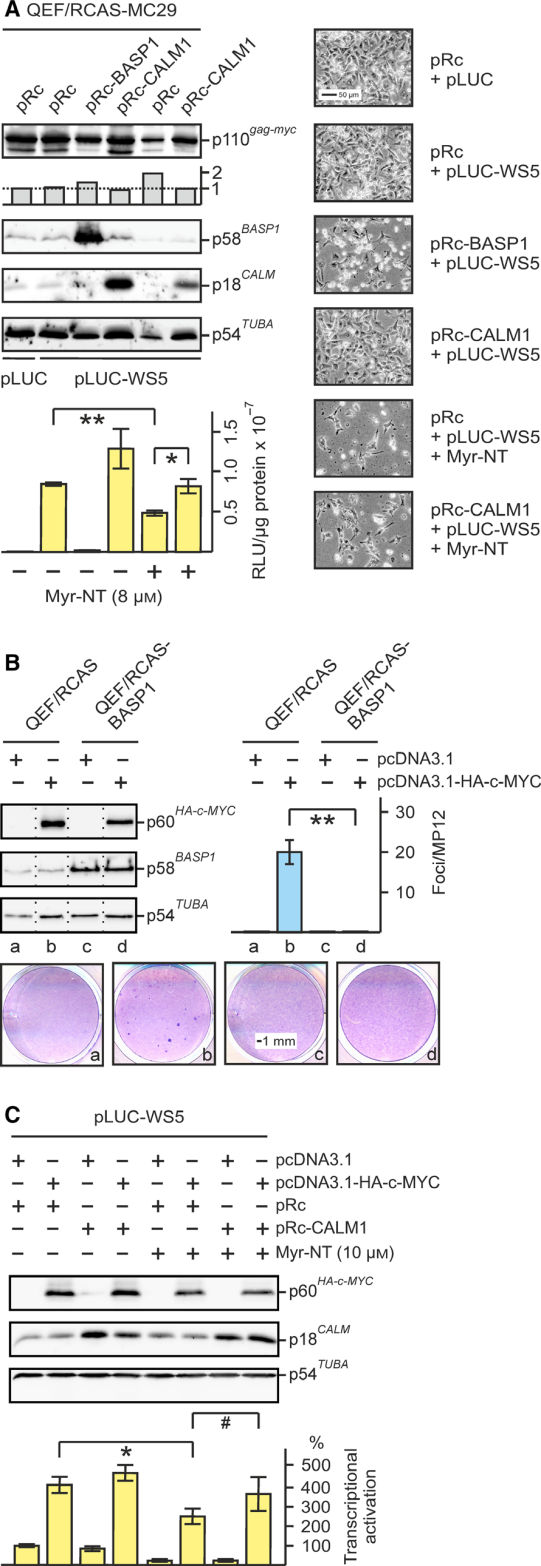
BASP1‐mediated inhibition of Myc‐induced transcriptional activation and restoration by ectopic CaM. (A) Transcriptional repression of the v‐Myc‐activated *WS5* promoter by BASP1 or the BASP1 peptide Myr‐NT, and recovery by ectopic CaM. The reporter construct pLUC‐WS5 specifying the Myc target *WS5* (Reiter *et al.*, 2007), or the empty vector (pLUC) (2 µg) was delivered into QEF/RCAS‐MC29 cells (4 × 10^6^) encoding Gag‐Myc (v‐Myc) *via* nucleofection together with 2 µg of the empty pRc vector (Rc), pRc‐BASP1 (BASP1), or pRc‐CALM1 (CaM). The Myr‐NT peptide was added as indicated to achieve a final concentration of 8 µm. Forty‐eight hours after nucleofection, LUC activities were measured (*n* = 2). Vertical error bars indicate standard deviations (SD) from triplicates. Statistical significance was assessed by using a paired Student’s *t*‐test (*P < 0.5, **P < 0.01; lower left panel). Protein expression was monitored by immunoblotting using antibodies directed against v‐Myc, BASP1, CaM, and α‐tubulin using equal amounts of protein extracts (upper left panel). Gag‐Myc expression normalized to the expression of α‐tubulin is visualized by vertical bars below the MYC blot, showing that relative Gag‐Myc amounts are not diminished in cells containing ectopic BASP1 or the Myr‐NT peptide. Micrographs of cells taken 48 h after nucleofection are shown on the right. (B) Cell transformation induced by human MYC and its inhibition by ectopic BASP1. QEF were transfected with pRCAS‐BASP1 or with the empty pRCAS vector, passaged four times, and then supertransfected with the eukaryotic expression vector pcDNA3.1 or pcDNA3.1‐HA‐c‐MYC. Cells were kept under agar overlay for 25 days and then stained with eosin methylene blue (lower panel). Foci were counted on MP12 dishes (*n* = 2). Vertical bars show standard deviations (SD) from triplicates. Statistical significance was assessed by using a paired Student’s *t*‐test (**P < 0.01; right panel). Proteins were analyzed by immunoblotting using equal amounts of cell extracts prepared 1 day after supertransfection and specific antisera directed against the HA tag, BASP1, or α‐tubulin (left panel). (C) Impact of the Myr‐NT peptide on human MYC‐dependent transcriptional activity analyzed in the absence and in the presence of ectopic CaM. The reporter construct pLUC‐WS5 was cotransfected into QT6 cells (2 × 10^5^) grown on MP24 wells together with the empty pcDNA3.1 (pc) or the pcDNA3.1‐HA‐c‐MYC (HA‐c‐MYC) vector, plus pRc‐CALM1 (CaM) or the empty pRc vector (Rc), all in the absence (−) or presence (+) of the transfected Myr‐NT peptide (10 µm). Equal aliquots (0.25 µg) of the individual plasmids were transfected, and 48 h after transfection, LUC activities were measured. The relative LUC activities calculated from three independent experiments (*n* = 3; ± SEM; *P < 0.05, ^#^ *P* = 0.07) are shown (lower panel). Protein expression was monitored by immunoblotting using antibodies directed against the HA‐tag (for HA‐c‐MYC), CaM, and α‐tubulin (upper panel).

To investigate whether the Myr‐NT peptide interferes with the transcriptional activation of target gene promoters by v‐Myc, the peptide and a reporter plasmid were transiently transfected by nucleofection into v‐*myc*‐transformed QEF/RCAS‐MC29 cells followed by LUC activity measurement. Plasmids encoding either BASP1 (pRc‐BASP1) or CaM (pRc‐CALM1) were included as control or as source for ectopic CaM, respectively (Fig. 6A). Most of the cells were efficiently transfected leading to high intracellular concentrations of BASP1, CaM, or the Myr‐NT peptide. In case of BASP1 or Myr‐NT, this led to a significant decrease in cell density of the v‐Myc‐transformed cells in contrast to the cells transfected with pRc‐CALM1, which were not affected (Fig. 6A). However, relative Gag‐Myc protein levels in these cells were not diminished as shown after normalization to α‐tubulin expression (Fig. 6A). Whereas pRc‐BASP1 efficiently suppressed v‐Myc‐mediated transcriptional activation of the *WS5* target gene promoter, pRc‐CALM1 led to an increase in transcriptional activation as reported previously (Hartl *et al.*, 2009; Raffeiner *et al.*, 2017). In the presence of the BASP1 ED peptide, the transcriptional activation potential was reduced to about 50% (Fig. 6A). Addition of ectopic CaM by pRc‐CALM1 restored the transcriptional activation, supporting the hypothesis that BASP1 inhibits v‐Myc by sequestering CaM.

We confirmed the key results obtained with the v‐Myc protein for the human MYC protein. An expression plasmid encoding MYC efficiently transformed QEF pretransfected with the empty RCAS vector, whereas QEF/RCAS‐BASP1 cells were resistant to transformation by MYC (Fig. 6B). To test for transcriptional activation, eukaryotic expression vectors encoding MYC or CaM were cotransfected into QT6 cells by the calcium phosphate method in the absence or presence of the BASP1 Myr‐NT peptide (Fig. 6C). Efficient transactivation of the *WS5* promoter by human MYC was slightly increased by ectopic CaM, whereas the Myr‐NT peptide repressed MYC‐mediated transcriptional activation to about 50%. On the other hand, ectopic CaM was able to restore this transcriptional repression (Fig. 6C) confirming the result obtained above (Fig. 6A).

### 3.6 BASP1‐mediated inhibition of MYC‐induced cell transformation is compensated by ectopic CaM expression

**Figure 7:**
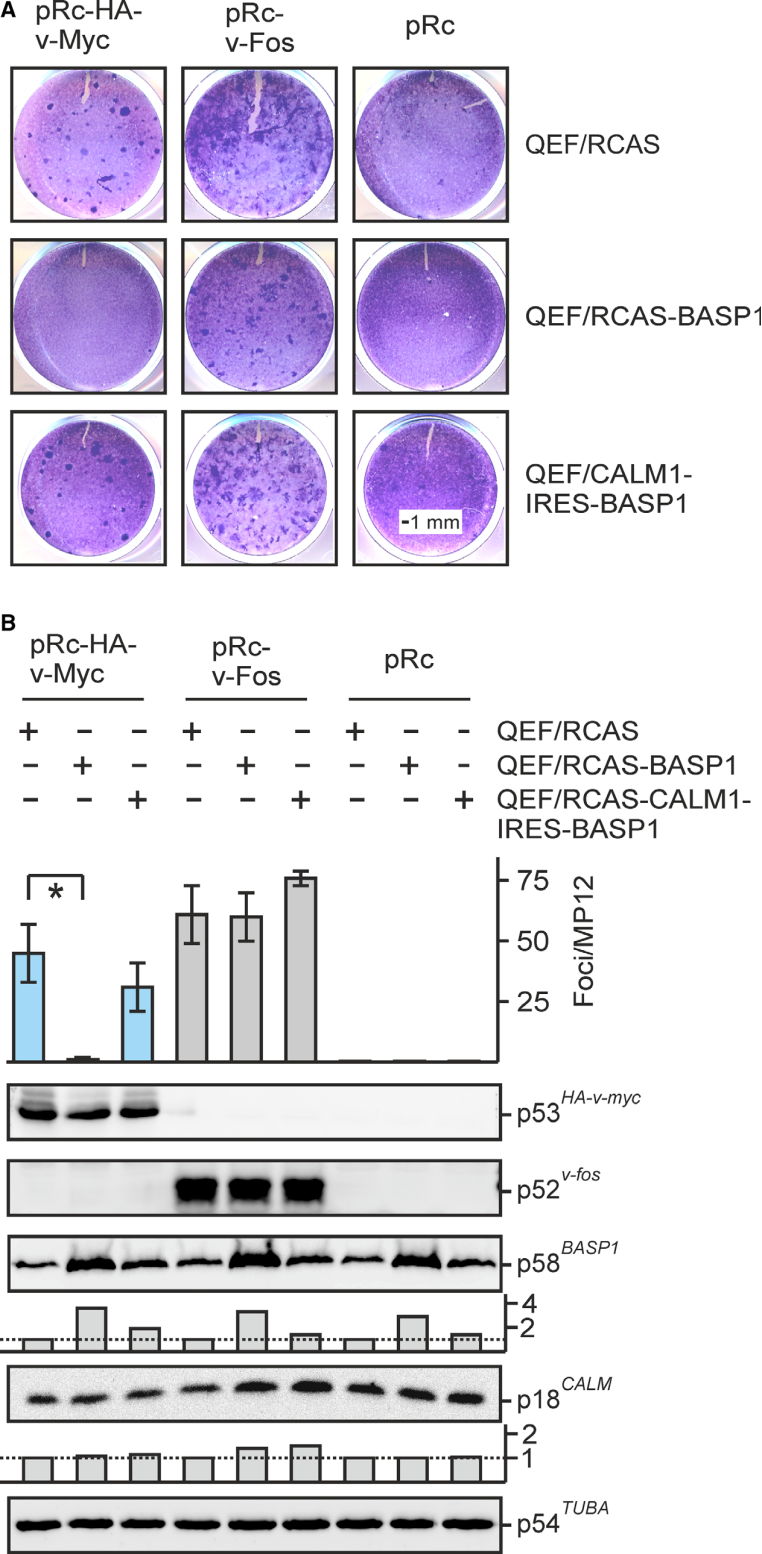
Restoration of v‐Myc‐induced cell transformation by ectopic CaM. (A) QEF were transfected with pRCAS‐BASP1, pRCAS‐CALM1‐IRES‐BASP1, or the empty pRCAS vector as in Fig. 1B, passaged four times, and then supertransfected with the eukaryotic expression vector pRc‐v‐Myc, pRc‐v‐Fos, or the empty pRc vector (Rc). Cells were kept under agar overlay for 19 days and then stained with eosin methylene blue. (B) Upper panel: Foci were counted on MP12 dishes (*n* = 2). Vertical bars show standard deviations (SD) from triplicates. Statistical significance was assessed by using a paired Student’s *t*‐test (*P < 0.05). Lower panel: Proteins were analyzed by immunoblotting using equal amounts of cell extracts prepared 1 day after supertransfection and specific antisera directed against v‐Myc, v‐Fos, BASP1, CaM, or α‐tubulin. Ectopic expression levels of BASP1 and CaM are visualized by vertical bars below the relevant blots.

**Figure 8:**
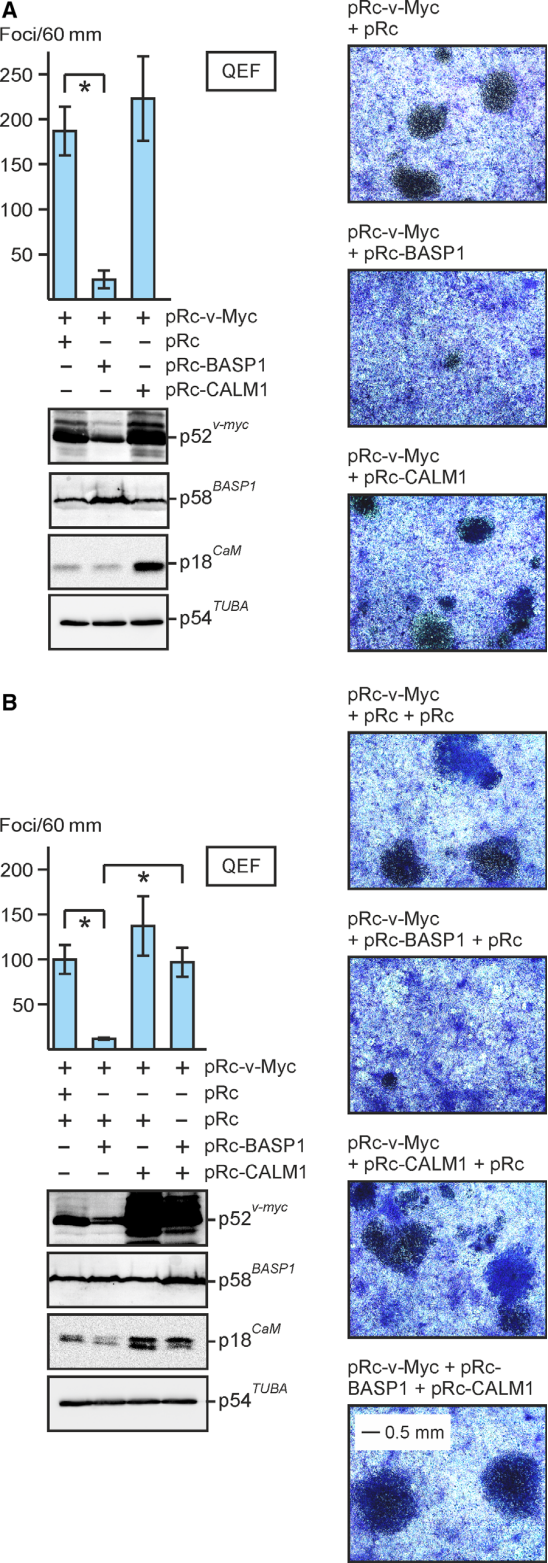
Cotransfection of eukaryotic expression vectors encoding v‐Myc, BASP1, and CaM into primary QEF. (A) Equal aliquots (6 µg) of pRc‐v‐Myc and pRc‐BASP1, or pRc‐v‐Myc and pRc‐CALM1, or pRc‐v‐Myc and the empty pRc vector were cotransfected into QEF. (B) Equal aliquots (4 µg) of the indicated plasmids were triple‐transfected in the shown combinations into QEF. Transfected cells were kept under agar overlay for 14 days and then stained with eosin methylene blue (right panels). Foci were counted on 60‐mm dishes (upper left panels). Vertical bars show standard deviations (SD) from triplicates (*n* = 2). Statistical significance was assessed by using a paired Student’s *t*‐test (*P < 0.05). Proteins were analyzed by immunoblotting using equal amounts of cell extracts prepared 1 day after transfection and specific antisera directed against v‐Myc, BASP1, CaM, or α‐tubulin (lower left panels).

In order to analyze whether CaM overexpression is also able to overcome BASP1‐mediated inhibition of v‐Myc‐induced cell transformation, QEF were transfected with RCAS, RCAS‐BASP1 (cf. Figs 1B and 6B), or bicistronic RCAS‐CALM1‐IRES‐BASP1 co‐expressing CaM and BASP1. Cells were then supertransfected with the eukaryotic expression vectors pRc‐v‐Myc, the empty pRc vector, or pRc‐v‐Fos as a control for the specificity of transformation inhibition (Fig. 7A). Ectopic BASP1 efficiently blocks cell transformation induced by v‐Myc but not by v‐Fos, although v‐Myc and v‐Fos proteins were expressed at comparable levels (Fig. 7B). In contrast, QEF/RCAS‐CALM1‐IRES‐BASP1 cells were efficiently transformed also by v‐Myc (Fig. 7B). CaM was only slightly overexpressed from the RCAS‐CALM1‐IRES‐BASP1 vector, but this was apparently sufficient to overcome the BASP1‐mediated v‐Myc inhibition. To demonstrate that low levels of ectopic CaM suffice to induce a specific biological effect, FLAG‐tagged CaM was co‐expressed with v‐Myc in QEF (Fig. S5) and tested for synergistic cell transformation as reported previously (Raffeiner *et al.*, 2017). The result shows that despite low overexpression, v‐Myc‐induced cell transformation was specifically enhanced in contrast to co‐expression with a FLAG‐tagged keratin control protein (KRN1) (Hartl *et al.*, 2001) with a size comparable to CaM (Fig. S5). To confirm that ectopic CaM compensates BASP1‐induced MYC inhibition, simultaneous transfections of pRc‐based plasmids encoding v‐Myc, BASP1, or CaM into primary QEF cells were performed yielding high ectopic protein levels, and cell transformation was then monitored by focus formation (Fig. 8). Cotransfections of pRc‐v‐Myc + pRc‐BASP1, or pRc‐v‐Myc + pRc‐CALM1, led to transformation repression or enhancement, respectively, as expected (Fig. 8A). Under these conditions, slight alterations in the v‐Myc protein levels were observed which were reduced in the presence of BASP1 or enhanced in the presence of CaM (Fig. 8A). This could be due to the observed effect that high BASP1 levels destabilize v‐Myc (cf. Fig. 4) whereas high CaM levels may have the opposite effect. Principally, the same effects on v‐Myc protein levels were observed in triple transfections, where overexpressed CaM even led to a dramatic increase in the amount of v‐Myc (Fig. 8B). Moreover, in these cells transfected with plasmids encoding v‐Myc, BASP1, and CaM, v‐Myc‐induced cell transformation was no longer inhibited by ectopic BASP1 (Fig. 8B) confirming the results obtained above that excess CaM compensates for the inhibitory effect of BASP1 on MYC‐induced oncogenesis.

## 4 Discussion

**Figure 9:**
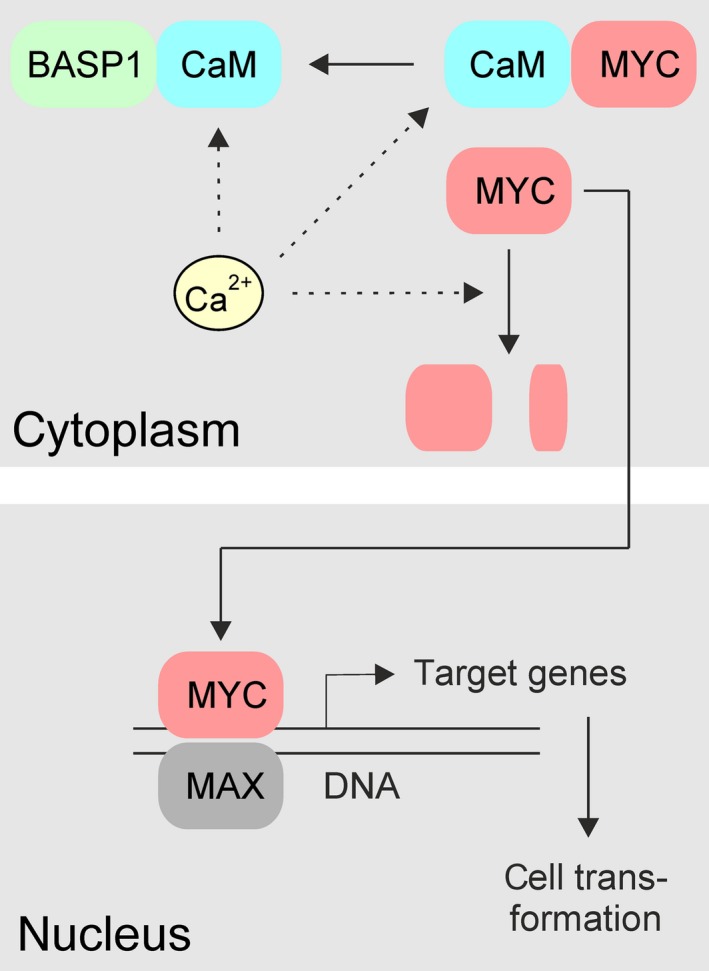
Schematic diagram showing the proposed mechanism of MYC inhibition by excess BASP1 protein competing for binding to CaM. Binding of CaM to BASP1 or MYC is Ca^2+^‐dependent (Maekawa *et al.*, 1993; Matsubara *et al.*, 2004; Raffeiner *et al.*, 2017). In addition to degradation by the ubiquitin–proteasome pathway, MYC is also specifically cleaved by calpains, Ca^2+^‐dependent proteases (Conacci‐Sorrell *et al.*, 2010; Farrell and Sears, 2014; Gregory and Hann, 2000). Interference of BASP1 with the MYC : CaM interaction leads to enhanced MYC degradation and less nuclear entry. Mitogenic signaling, amplification, translocation, or retroviral transduction leads to *MYC* hyperactivation and subsequent deregulation of numerous transcriptional targets including strong repression of the tumor suppressor *BASP1* gene (Hartl *et al.*, 2009).

MYC primarily functions as a transcriptional activator or amplifier. However, in the case of certain specific genes, it has also been shown to display transcriptional repressor activity (Conacci‐Sorrell *et al.*, 2014; Dang, 2014; Eilers and Eisenman, 2008; Hartl, 2016; Stefan and Bister, 2017; Wolf *et al.*, 2015). In a screen for MYC target genes using cell lines conditionally transformed by doxycycline‐controlled v‐*myc* alleles, we have previously isolated the *BASP1* gene based on its nearly complete transcriptional suppression in v‐Myc‐transformed cells (Hartl *et al.*, 2009). Strikingly, ectopic expression of *BASP1* rendered fibroblasts resistant to subsequent transformation by v‐Myc and strongly attenuated the transformed phenotype and viability of cells with established v‐Myc transformation. In addition, ectopic BASP1 interfered with the transcriptional regulation of known MYC target genes. We concluded that downregulation of the *BASP1* gene is a necessary event in MYC‐induced oncogenesis and that the BASP1 protein may act as a potential tumor suppressor (Hartl *et al.*, 2009). Mutational analysis revealed that the basic N‐terminal ED encompassing a myristoylation site, a CaM‐binding domain, and a putative nuclear localization signal is essential for the interference of BASP1 with MYC‐induced cell transformation (Hartl *et al.*, 2009) (cf. Fig. S1). BASP1 and MYC do not interact directly (Hartl *et al.*, 2009) (cf. Fig. 3), but we recently reported that all MYC variants (c‐MYC, v‐Myc, N‐MYC, L‐MYC) bind to CaM (Raffeiner *et al.*, 2017), possibly indicating that the antagonistic functions of BASP1 and MYC may involve their shared binding partner CaM. CaM strongly binds to v‐Myc but only weakly, or not at all to other oncogenic transcription factors or protein kinases indicating a special affinity for MYC (cf. Fig. 1). We also show here that ectopic BASP1 expression interferes with the v‐Myc : CaM interaction and that this inhibition correlates with decreased v‐Myc protein stability (cf. Figs 2, 3, 4). Moreover, ectopic CaM can compensate for the inhibitory BASP1 effects on transcriptional regulation and cell transformation by v‐Myc (cf. Figs 6, 7, 8). MYC proteins are predominantly localized in the nucleus to execute transcriptional regulation, but a substantial fraction is also present in the cytoplasm (Conacci‐Sorrell *et al.*, 2010; Raffeiner *et al.*, 2017). In this cell compartment, proteolytic cleavage of MYC by calpains leads to a carboxyl‐terminally truncated form termed MYC‐nick which promotes α‐tubulin acetylation and cell differentiation (Conacci‐Sorrell *et al.*, 2010). A discrete cleavage of the Gag‐Myc (v‐Myc) protein was also observed in cells simultaneously expressing v‐Myc and BASP1 (cf. Fig. 4A) (Raffeiner *et al.*, 2017). In the absence of BASP1, the v‐Myc protein is quite stable presumably due to mutation of a critical threonine residue (corresponding to T58 in human MYC) in the amino‐terminal MYC box I (Stefan and Bister, 2017), rendering v‐Myc resistant toward GSK3β‐mediated phosphorylation and subsequent ubiquitin–proteasome‐mediated degradation as it occurs for c‐MYC (Farrell and Sears, 2014; Gregory and Hann, 2000; Stefan and Bister, 2017). This may explain the prolonged v‐Myc stability after CHX treatment in the absence of BASP1 (cf. Fig. 4A, Fig. S2). Based on our previous (Hartl *et al.*, 2009; Raffeiner *et al.*, 2017) and the current results reported here, we suggest that BASP1 competes with v‐Myc for the calcium sensor CaM leading to decreased protein stability and interference with the transcriptional and oncogenic functions of MYC (Fig. 9). The mutual interference between v‐Myc and BASP1 could also elucidate why v‐Myc downregulates the *BASP1* gene already during the initiation phase of cell transformation as reported recently (Hartl *et al.*, 2009; Valovka *et al.*, 2013). The functional connection between MYC, BASP1, and CaM may also explain decreased CaM levels in cells with ectopic MYC and increased CaM levels in cells with ectopic BASP1 (cf. Figs 2A, 3A, and 6C, Fig. S2). Depending on the amount of BASP1 within a cell, CaM levels could vary in order to guarantee a minimal concentration of free CaM required for multiple cellular functions, which is not sequestered by BASP1. Because MYC downregulates BASP1, less CaM is required when compared to BASP1‐overexpressing cells, in which increased CaM expression is necessary to compensate for BASP1‐bound CaM.

The *BASP1* gene is downregulated in most mammalian cancers, and tumor‐suppressive functions of BASP1 were observed in several human cancer models (Guo *et al.*, 2016; Marsh *et al.*, 2017; Zhang *et al.*, 2019; Zhou *et al.*, 2019; Zhou *et al.*, 2018). This corroborates our original finding that BASP1 strongly interferes with v‐Myc‐induced oncogenicity and displays properties of a tumor suppressor (Hartl *et al.*, 2009). In multiple carcinoma, melanoma, and leukemia cells, *BASP1* transcription is silenced by promoter methylation (Kaehler *et al.*, 2015; Moribe *et al.*, 2008; Zhou *et al.*, 2018), a typical DNA modification in the regulatory regions of tumor suppressors in cancer. An important function of BASP1 is to act as a transcriptional cosuppressor of the Wilms’ tumor suppressor protein WT1, converting the WT1 oncoprotein into a tumor suppressor (Carpenter *et al.*, 2004; Goodfellow *et al.*, 2011; Toska *et al.*, 2012; Toska *et al.*, 2014). WT1 is a gene regulator important for cell growth, apoptosis, and differentiation, and multiple genes are regulated by WT1. Interestingly, one of the WT1 targets is *MYC*, which is activated by WT1 but suppressed by a WT1 : BASP1 complex (Goodfellow *et al.*, 2011; Green *et al.*, 2009; Han *et al.*, 2004; Wu *et al.*, 2015). Recent studies showed that WT1 is activated in pancreatic cancer and that patients with elevated BASP1 levels have a significantly better prognosis than individuals whose cancer cells contain no BASP1 but high WT1 levels (Zhou *et al.*, 2019).

*BASP1* may represent one of the essential MYC target genes playing a direct role in cell transformation and maintenance of the transformed state. For CaM binding and inhibition of v‐Myc‐induced cell transformation, the amino‐terminal myristoylated BASP1 ED encompassing 11 amino acid residues is sufficient (Hartl *et al.*, 2009). In fact, mutational analysis of this highly conserved domain has revealed that amino acid residues essential for CaM binding (Matsubara *et al.*, 2004) are also critical for the transformation inhibition potential (Hartl *et al.*, 2009) (cf. Fig. S1). Accordingly, a myristoylated peptide representing the BASP1 ED interferes with the growth of v‐Myc‐transformed cells and of human cancer cell lines containing elevated *MYC* levels (cf. Fig. 5, Figs S3 and S4). Therefore, this small interfering peptide could be used as a template for the design of therapeutic peptides to inhibit human cancers displaying high MYC expression. The design of BASP1 therapeutic peptides would have to include engineering of cell‐penetrating capacities, similar to the successful application of cell‐penetrating peptides based on the dominant‐negative MYC inhibitor Omomyc in various MYC‐related cancer models including triple‐negative breast cancer cells (Beaulieu *et al.*, 2019; Wang *et al.*, 2019).

The BASP1 effector peptide does not directly bind to MYC but acts more like a CaM antagonist, similar to the compounds TFP, *N‐*(6‐aminohexyl)‐5‐chloro‐1‐naphthalenesulfonamide hydrochloride (W‐7), or TMX. TFP or W‐7 is small organic molecule binding tightly to the hydrophobic CaM pockets, similar to the BASP1 ED peptide (Matsubara *et al.*, 2004; Osawa *et al.*, 1998; Vandonselaar *et al.*, 1994). Interestingly, the phenothiazine derivative TFP, representing a U.S. Food and Drug Administration‐approved antipsychotic drug, inhibits proliferation and the transformed phenotype of v‐*myc*‐transformed cells (cf. Fig. 5A) and thus has anti‐oncogenic properties. In fact, TFP also interferes with the invasive growth of multiple human cell types derived from lung and breast cancer, hepatocellular carcinoma, or T‐cell lymphoma. TFP offers a limited cytotoxic profile and is therefore discussed as potential available drug in cancer therapy (Feng *et al.*, 2018; Pulkoski‐Gross *et al.*, 2015). In glioblastoma, TFP suppresses tumor cell proliferation and invasion *in vitro* and *in vivo*. Thereby, TFP binds to CaM and causes its dissociation from inositol 1,4,5‐triphosphate receptor leading to channel opening and increase in intracellular calcium levels (Kang *et al.*, 2017). The anti‐estrogen TMX antagonistically binds to the estrogen receptor (ER) and to CaM in a calcium‐dependent manner (Lopes *et al.*, 1990). Both TMX and TFP induce apoptosis in cholangiocarcinoma (Pawar *et al.*, 2009), and it is interesting that the CaM interactor BASP1 was reported to enhance the antitumorigenic effect of TMX in breast cancer (Marsh *et al.*, 2017). In addition, the androgen receptor (AR) interacts with CaM (Cifuentes *et al.*, 2004). Intriguingly, W7 and TFP enhance apoptosis in prostate cancer cells by promoting AR proteolysis upon liberation from CaM (Sivanandam *et al.*, 2011). This is reminiscent of the observation reported here that inhibition of MYC‐induced cell transformation by the CaM interactor BASP1 is accompanied by a decrease in MYC protein stability (cf. Figs 4 and 9, Fig. S2).

## 5 Conclusion

The *MYC* gene is a major cancer driver, and deregulation of *MYC* expression is a hallmark of the majority of human tumors. Therefore, *MYC* has become an obvious but also difficult therapeutic target for cancer therapy. As for many transcription factors, direct specific inhibition of the MYC protein remains a challenging task. Nevertheless, several strategies interfering with *MYC* gene transcription or MYC protein function have been applied so far. We previously identified the *BASP1* gene as a negative transcriptional target of MYC. *BASP1* overexpression strongly interferes with *MYC*‐induced oncogenesis and the BASP1 protein displays properties of a tumor suppressor, also in human cancer. BASP1 is a CaM‐binding protein, and we reported recently that MYC proteins also bind tightly to CaM. In the current work, we present evidence for a functional connection between MYC, BASP1, and CaM. We propose that BASP1 competes with MYC for CaM binding leading to MYC protein destabilization as the molecular mechanism of BASP1‐induced MYC inhibition. Accordingly, the structure of the BASP1 ED could be used as template for the design of small molecules or peptides in cancer drug development.

## Conflict of interest

The authors declare no conflict of interest.

## Author contributions

MH and KB conceived research. MH, KP, AN, and PR performed experiments and analyzed data. MH and KB wrote the paper.

## Supporting information


**Fig. S1.** Mutational analysis of the BASP1 effector domain to test critical amino acid residues required for CaM binding, and for suppression of cell transformation triggered by v‐Myc.

**Fig. S2.** Stability of the Gag‐Myc protein in the presence of BASP1, and upon pharmacological CaM inhibition.

**Fig. S3.** Inhibition of v‐Myc‐triggered cell proliferation and of v‐Myc : CaM binding by the BASP1 effector domain.

**Fig. S4.** Inhibitory effect of the BASP1 effector domain on the proliferation of human leukemia cell lines with high endogenous *MYC* levels.

**Fig. S5.** Specific enhancement of v‐Myc‐induced cell transformation by ectopic CaM.
