# Supplementary figures and images for: The brain acid‐soluble protein 1 (BASP1) interferes with the oncogenic capacity of MYC and its binding to calmodulin
Source: Mol Oncol. 2020 Jan 30;14(3):625–44. doi: 10.1002/1878-0261.12636 (PMC7053243; doi:10.1002/1878-0261.12636)

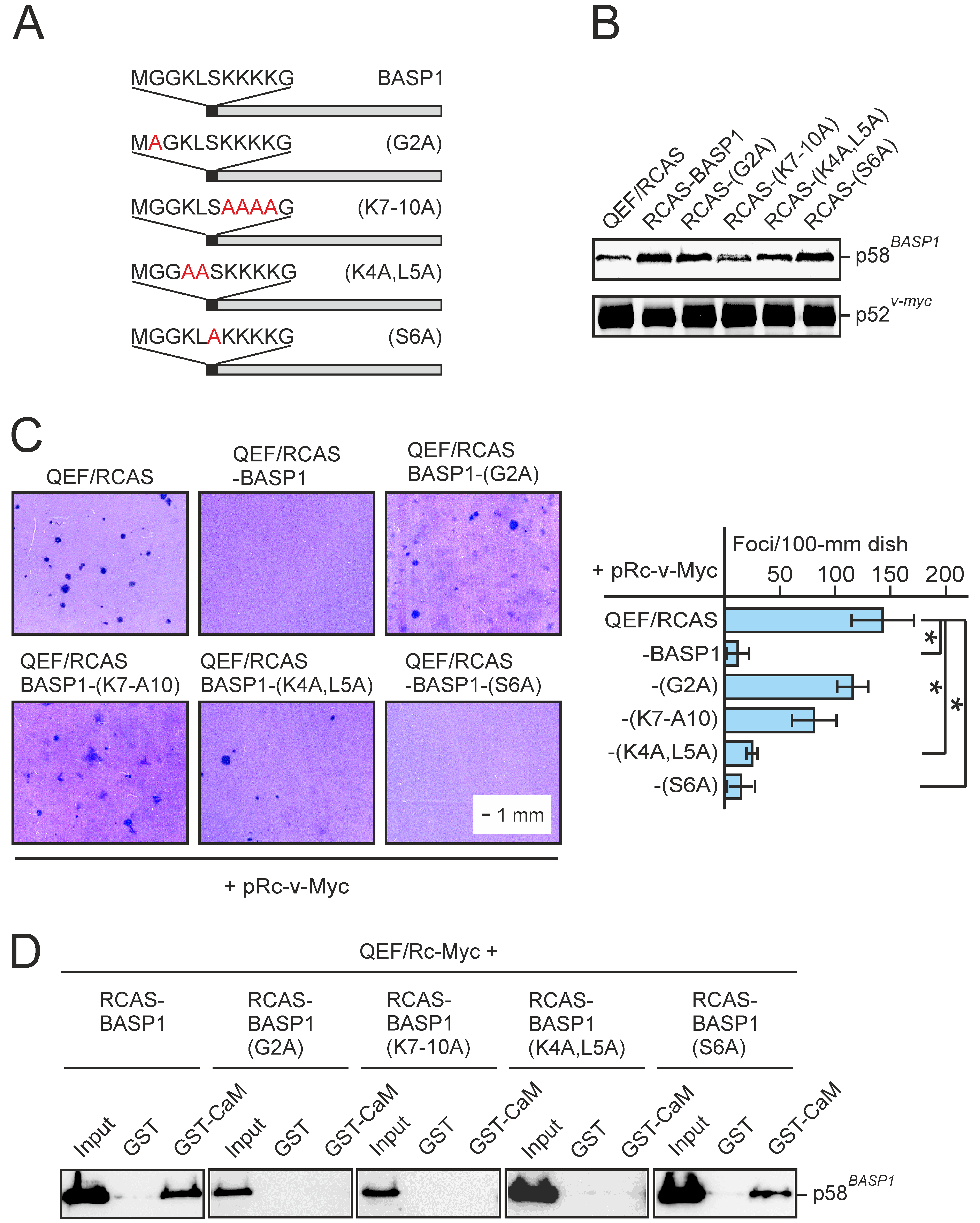

Supplement: Supplementary file 1 — Fig. S1. Mutational analysis of the BASP1 effector domain to test critical amino acid residues required for CaM binding, and for suppression of cell transformation triggered by v‐Myc. [file MOL2-14-625-s001.tif]

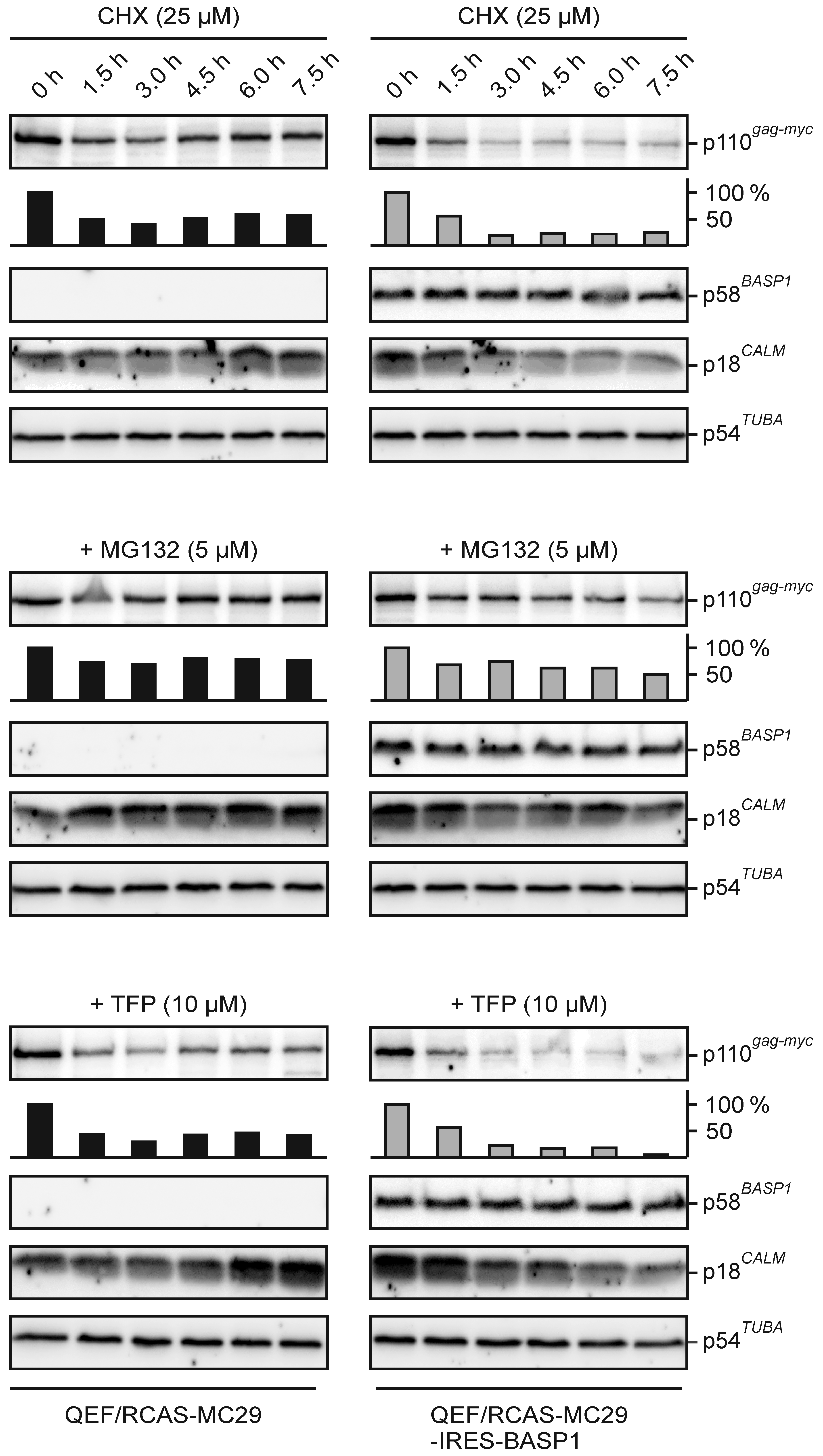

Supplement: Supplementary file 2 — Fig. S2. Stability of the Gag‐Myc protein in the presence of BASP1, and upon pharmacological CaM inhibition. [file MOL2-14-625-s002.tif]

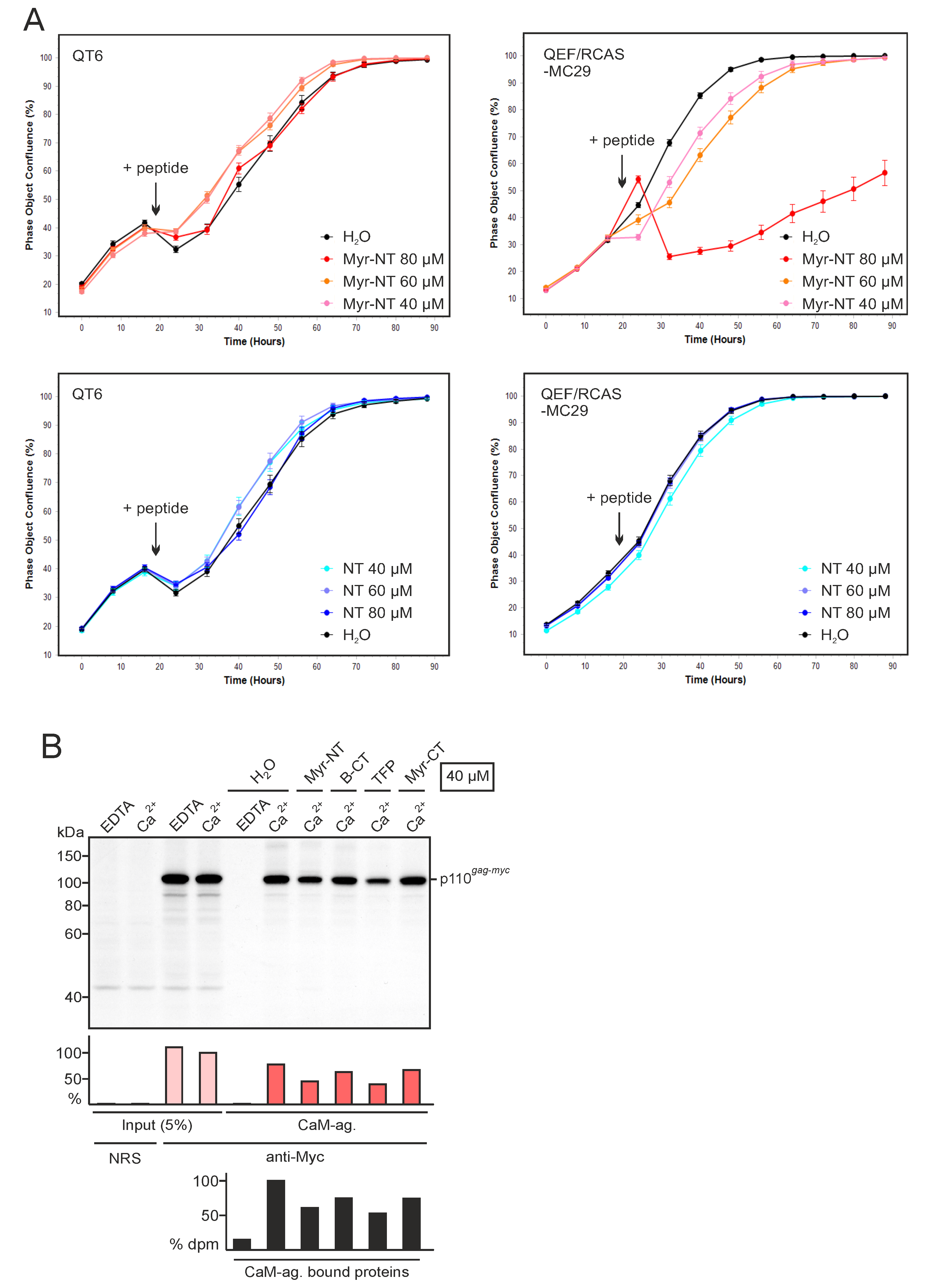

Supplement: Supplementary file 3 — Fig. S3. Inhibition of v‐Myc‐triggered cell proliferation and of v‐Myc : CaM binding by the BASP1 effector domain. [file MOL2-14-625-s003.tif]

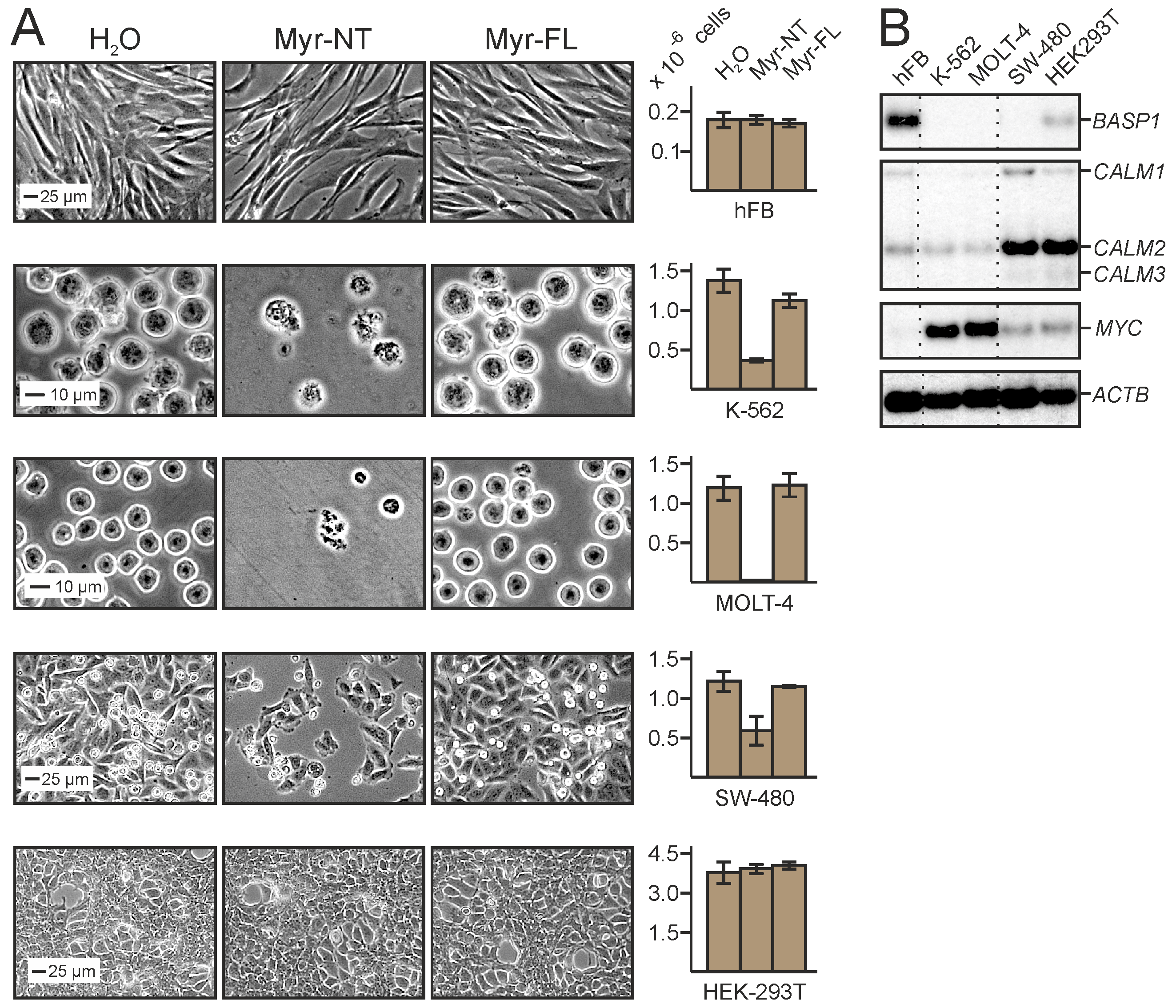

Supplement: Supplementary file 4 — Fig. S4. Inhibitory effect of the BASP1 effector domain on the proliferation of human leukemia cell lines with high endogenous MYC levels. [file MOL2-14-625-s004.tif]

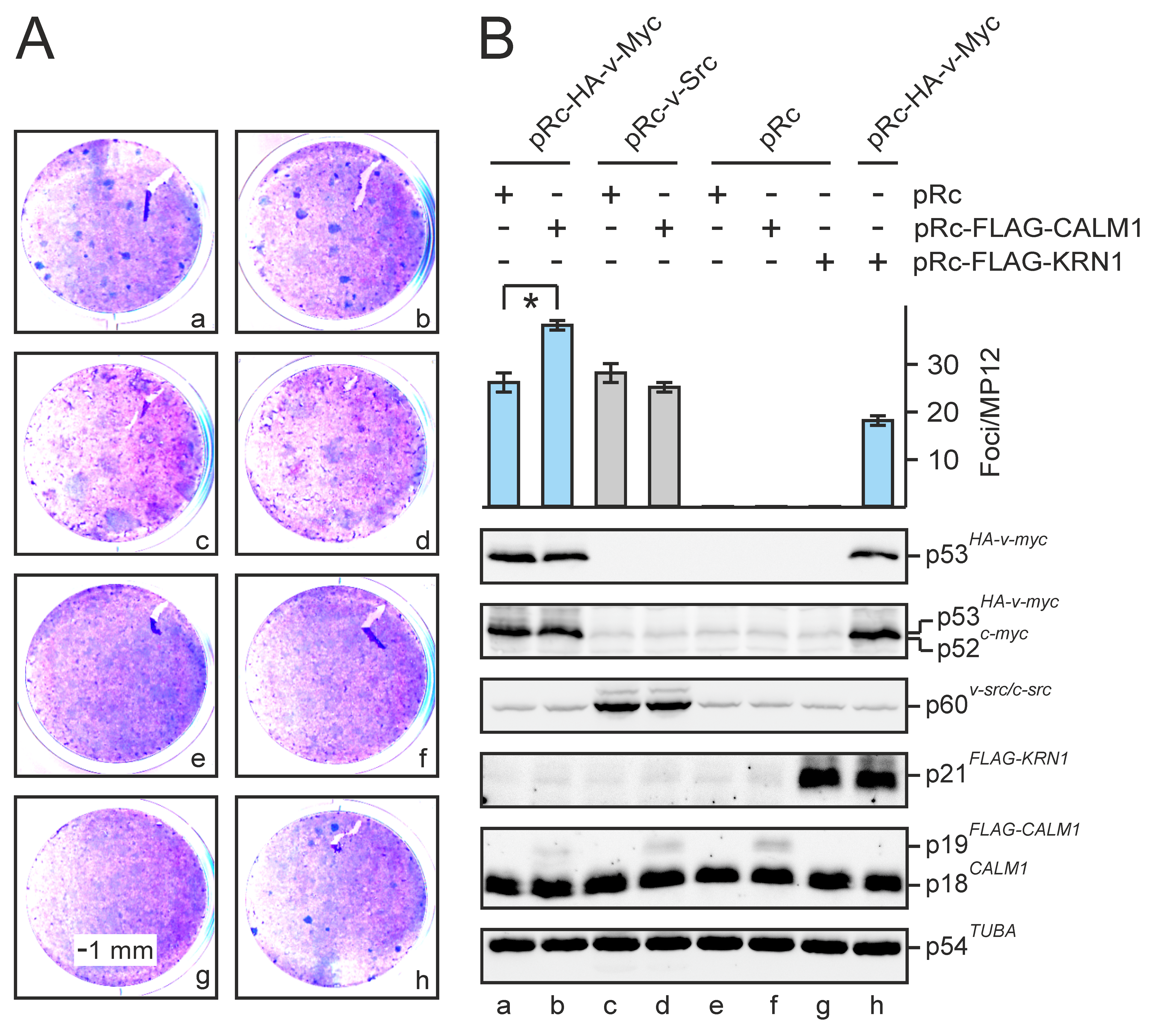

Supplement: Supplementary file 5 — Fig. S5. Specific enhancement of v‐Myc‐induced cell transformation by ectopic CaM. [file MOL2-14-625-s005.tif]
