# Supplementary material for: The brain acid‐soluble protein 1 (BASP1) interferes with the oncogenic capacity of MYC and its binding to calmodulin
Source: Mol Oncol. 2020 Jan 30;14(3):625–44. doi: 10.1002/1878-0261.12636 (PMC7053243; doi:10.1002/1878-0261.12636)
Supplement: Supplementary file 6 [file MOL2-14-625-s006.docx]

*Revised manuscript MOLONCOL-19-0603.R3*

**Supporting information (Hartl *et al*.)**

**Figure S1.** Mutational analysis of the BASP1 effector domain to test critical amino acid residues required for CaM binding (Matsubara *et al*., 2004), and for suppression of cell transformation triggered by v-Myc. (A) Schematic diagram of wild type and mutant BASP1 proteins. Engineered amino acid substitutions (red) are indicated. (B) Quail embryo fibroblasts (QEF) were transfected with pRCAS-BASP1 or with the empty pRCAS vector, passaged four times (QEF/RCAS, QEF/BASP1), and then supertransfected with the eukaryotic expression vector pRc-v-Myc encoding the v-*myc* oncogene. Proteins were analyzed by immunoblotting using equal amounts of cell extracts prepared two days after supertransfection and specific antisera directed against BASP1 and Myc. (C) Cells were kept under agar overlay for 14 d, and then stained with eosin methylene blue (left panel). Foci were counted on 100-mm dishes (n=3). Horizontal bars show standard deviations (SD) from triplicates (right panel). Statistical significance was assessed by using a paired Student t-test (*P < 0.05). (D) Protein pull-down of BASP1 and BASP1 mutants using a glutathione-S-transferase (GST)-calmodulin (CaM) fusion protein coupled to glutathione-sepharose beads. Eluted proteins were resolved by SDS-PAGE, immunoblotted using a BASP1-specific antibody, and detected by enhanced chemoluminescence (ECL). Blots were exposed for 20 min to verify the weak interaction between CaM and the K4A/L5A mutant.

**Figure S2.** Stability of the Gag-Myc protein in the presence of BASP1, and upon pharmacological CaM inhibition. Equal numbers of QEF/RCAS-MC29 (1.0 × 10^5^) and QEF/RCAS-MC29-IRES-BASP1 (1.5 × 10^5^) cells were seeded onto MP12 wells and incubated in the presence of 25 µM cycloheximide (CHX), and with the proteasome inhibitor MG-132 (5 µM), or the CaM inhibitor trifluoperazine (TFP). Cell extracts were prepared after the indicated time points (0-7.5 h). Proteins were analyzed by immunoblotting using equal amounts of cell extracts and specific antisera directed against v-Myc, BASP1, CaM, or α-tubulin. Relative quantifications of Gag-Myc expression are given below the MYC blots. The presence of TFP leads to a further decrease in Gag-Myc stability.

**Figure S3.** Inhibition of v-Myc-triggered cell proliferation and of v-Myc:CaM binding by the BASP1 effector domain. (A) Proliferation kinetics of QT6 (left panels) and QEF/RCAS-MC29 (right panels) cells in the presence of various concentrations (40-80 µM) of the N-terminal BASP1 effector domain peptide (Myr-NT) which binds to CaM (Matsubara *et al.*, 2004), or a non-myristoylated derivate (NT). QEF/RCAS-MC29 cells expressing the Gag-Myc fusion protein (5 × 10^3^) or, as a control, chemically transformed QT6 cells (2 × 10^4^) were seeded onto 96-well cell culture plates in 100 µl culture medium. The next day, the peptides were added as described (Raffeiner *et* *al*., 2014) by replacing the medium with 50 µl of medium without serum containing BASP1 peptides at the indicated final concentrations. Cells were then incubated at 37°C. After 8 h, 50 µl of medium containing 2x serum and the final concentrations of the peptides was added. Cell densities were measured every 8 h over a 3-day time period using an Incucyte live cell analysis system. Cells without treatment (H_2_O) were used as reference. (B) Impairment of the v‑Myc:CaM interaction by trifluoperazine (TFP), or by the Myr-NT peptide. For specific pull-down of the Gag-Myc hybrid protein bound to CaM-agarose, lysates were prepared under native conditions from metabolically [^35^S]-methionine-labelled QEF/RCAS-MC29 cells. Lysates were loaded onto CaM-agarose beads in the presence of CaCl_2_ (0.5 mM) or EDTA (1 mM), and Myr-NT (40 µM) or TFP (40 µM). Unrelated myristoylated or non-myristoylated peptides (Myr-CT, B‑CT) were used as a control. Bound proteins were eluted under denaturing conditions, and subjected to immunoprecipitation using polyclonal antibodies directed against Myc, or normal rabbit serum (NRS). As input control, 5% of the lysates were used for immunoprecipitation. Proteins were resolved by SDS-PAGE (10% w/v) and radioactive signals detected on a bioimager. Positions of protein size markers are indicated in the margin. Relative quantifications of input (*pink*) and pull-down (*red*) signals are shown below. The total amounts of radiolabeled proteins bound to CaM prior to immunoprecipitation are depicted as black bars in the lower panel (dpm, distintegrations per minute).

**Figure S4.** Inhibitory effect of the BASP1 effector domain on the proliferation of human leukemia cell lines with high endogenous *MYC* levels. (A) Cells were treated with a myristoylated peptide encompassing the highly conserved BASP1 effector domain (Myr‑NT), or with a myristoylated control peptide (Myr-FL) in ECB buffer as described (Hart *et al*., 2014) to achieve final concentrations of 80 µM, and incubated at 37°C for 2 days. Afterwards, cell numbers were determined (n = 2). Phase contrast micrographs (left panel) and cell numbers (right panel) of human fibroblasts (hFB), K‑562, MOLT-4, SW-480, and HEK-293T cells. Vertical bars show standard deviations (SD) from triplicates. (B) Northern analysis of RNAs from the tested cells using probes specific for *BASP1*, the three calmodulin transcripts (*CALM1‑3*), *MYC*, and actin beta (*ACTB*) as loading control. The dotted lines mark splicing sites in the blot images, from which two lanes have been removed.

**Figure S5.** Specific enhancement of v-Myc-induced cell transformation by ectopic CaM. (A) Equal aliquots (1 µg) of pRc-HA-v-Myc, pRc-v-Src or the empty pRc vector were transfected into QEF together with the empty pRc vector, or pRc derived constructs encoding tagged CaM (pRc-FLAG-CALM1), or human keratin-associated protein 5.9 (pRc-FLAG-KRN1) into QEF. Transfected cells were kept under agar overlay for 14 d, and then stained with eosin methylene blue. (B) Foci were counted on MP-12 dishes (upper panel). Vertical bars show standard deviations (SD) from triplicates (n = 2). Statistical significance was assessed by using a paired Student t‑test (*P < 0.05). Proteins were analyzed by immunoblotting using equal amounts of cell extracts prepared 1 d after transfection and specific antisera directed against HA, v-Myc, v-Src, FLAG, CaM, or α-tubulin (lower panel).

**References**

Hart, J.R., Garner, A.L., Yu, J., Ito, Y., Sun, M., Ueno, L., Rhee, J.K., Baksh, M.M., Stefan, E., Hartl, M., Bister, K., Vogt, P.K., Janda, K.D., 2014. Inhibitor of MYC identified in a Krohnke pyridine library. Proc Natl Acad Sci U S A 111, 12556-12561.

Matsubara, M., Nakatsu, T., Kato, H., Taniguchi, H., 2004. Crystal structure of a myristoylated CAP-23/NAP-22 N-terminal domain complexed with Ca2+/calmodulin. EMBO J 23, 712-718.

Raffeiner, P., Röck, R., Schraffl, A., Hartl, M., Hart, J.R., Janda, K.D., Vogt, P.K., Stefan, E., Bister, K., 2014. In vivo quantification and perturbation of Myc-Max interactions and the impact on oncogenic potential. Oncotarget 5, 8869-8878.
